# Comparative transcriptome analysis of soybean response to bean pyralid larvae

**DOI:** 10.1186/s12864-017-4256-7

**Published:** 2017-11-13

**Authors:** Weiying Zeng, Zudong Sun, Zhaoyan Cai, Huaizhu Chen, Zhenguang Lai, Shouzhen Yang, Xiangmin Tang

**Affiliations:** Guangxi Academy of Agricultural Sciences, Nanning, Guangxi 530007 China

**Keywords:** Soybean, Bean pyralid, Transcriptome sequencing, Differentially expressed genes (DEGs)

## Abstract

**Background:**

Soybean is one of most important oilseed crop worldwide, however, its production is often limited by many insect pests. Bean pyralid is one of the major soybean leaf-feeding insects in China. To explore the defense mechanisms of soybean resistance to bean pyralid, the comparative transcriptome sequencing was completed between the leaves infested with bean pyralid larvae and no worm of soybean (Gantai-2-2 and Wan82–178) on the Illumina HiSeq™ 2000 platform.

**Results:**

In total, we identified 1744 differentially expressed genes (DEGs) in the leaves of Gantai-2-2 (1064) and Wan82–178 (680) fed by bean pyralid for 48 h, compared to 0 h. Interestingly, 315 DEGs were shared by Gantai-2-2 and Wan82–178, while 749 and 365 DEGs specifically identified in Gantai-2-2 and Wan82–178, respectively. When comparing Gantai-2-2 with Wan82–178, 605 DEGs were identified at 0 h feeding, and 468 DEGs were identified at 48 h feeding. Gene Ontology (GO) annotation analysis revealed that the DEGs were mainly involved in the metabolic process, single-organism process, cellular process, responses to stimulus, catalytic activities and binding. Pathway analysis showed that most of the DEGs were associated with the plant-pathogen interaction, phenylpropanoid biosynthesis, phenylalanine metabolism, flavonoid biosynthesis, peroxisome, plant hormone signal transduction, terpenoid backbone biosynthesis, and so on. Finally, we used qRT-PCR to validate the expression patterns of several genes and the results showed an excellent agreement with deep sequencing.

**Conclusions:**

According to the comparative transcriptome analysis results and related literature reports, we concluded that the response to bean pyralid feeding might be related to the disturbed functions and metabolism pathways of some key DEGs, such as DEGs involved in the ROS removal system, plant hormone metabolism, intracellular signal transduction pathways, secondary metabolism, transcription factors, biotic and abiotic stresses. We speculated that these genes may have played an important role in synthesizing substances to resist insect attacks in soybean. Our results provide a valuable resource of soybean defense genes that will benefit other studies in this field.

**Electronic supplementary material:**

The online version of this article (10.1186/s12864-017-4256-7) contains supplementary material, which is available to authorized users.

## Background

Soybean (*Glycine max* (L.) Merr.) is the largest oil crop worldwide, and is widely used in the production of food, feed, industrial products and other sideline fields [1]. However, there have been large increases in soybean production costs due to pests. Bean pyralid (*Lamprosema indicate* (Fabricius)) is one of the major leaf-feeding insects that affects soybean crops in central and southern China, the larvae lurk inside soybean leaves, cause leaf curling and feed on leaf tissues, affecting photosynthesis. Therefore, the plants cannot grow normally [2]. So bean pyralid is different from other leaf-feeding insects with chewing mouthparts which cause holes or incisions by means of encroachment [3]. In the soybean-producing areas of southern China, many generations of bean pyralids may appear in 1 year. In serious pest-damaged years, only veins and petioles will be left, causing serious yield losses [4]. Sun et al. and Long et al. evaluated rolled leaflet number and larva number could be used as an evaluation index for bean pyralid in soybean, and screened the highly resistant line Gantai-2-2 and the highly sensitive line Wan 82–178 [4–6]. Two indicators of resistance to bean pyralid, rolled leaflet number and rolled leaflet percentage, were a significantly positively correlated with the pubescence angle, length on leaf blade, angle on petiole and a significantly negatively correlated with the pubescence density on leaf blade, but on correlation with pubescence tip shape was observed [7]. Xing et al. and Li et al. showed that soybean resistance to bean pyralid accords with two or three major genes and polygene, 81–92% of the phenotypic variation was accounted for by additive quantitative trait locus (QTL) (27–43%), epistatic QTL pairs (5–13%) and collective unmapped minor QTL (38–58%) [8–10]. The contents of soluble sugar, superoxide dismutase (SOD), polyphenol oxidase (PPO), jasmonate (JA) and abscisic acid (ABA) are significantly increased after bean pyralid feeding [11]. However, the results of comparative transcriptome research which has focused on soybeans’ resistance to bean pyralid has not yet been made available. This is the first study of soybean transcriptome in response to bean pyralid feeding.

Transcriptome sequencing has become an important method for gene expression analysis, differentially expressed genes (DEGs) selection, functional gene mining, and genetic evolution analysis. The soybean genome was released in 2010 [12]. Based on the soybean genome data and transcriptome technology, can be better to examine all the transcription reactions, structural functions, and transcriptional regulation of resistant soybean varieties at the overall level. In the present paper, we tried to find important DEGs and metabolism pathways might related to the soybean in response to bean pyralid larvae through the comparative transcrptome analysis between the leaves infested with bean pyralid larvae and no worm of soybean using the Illumina HiSeq™ 2000 platform. Our results provide a valuable resource of soybean defense genes that will benefit other studies in this field.

## Results

### Transcriptome sequencing and sequence alignment

**Fig. 1:**
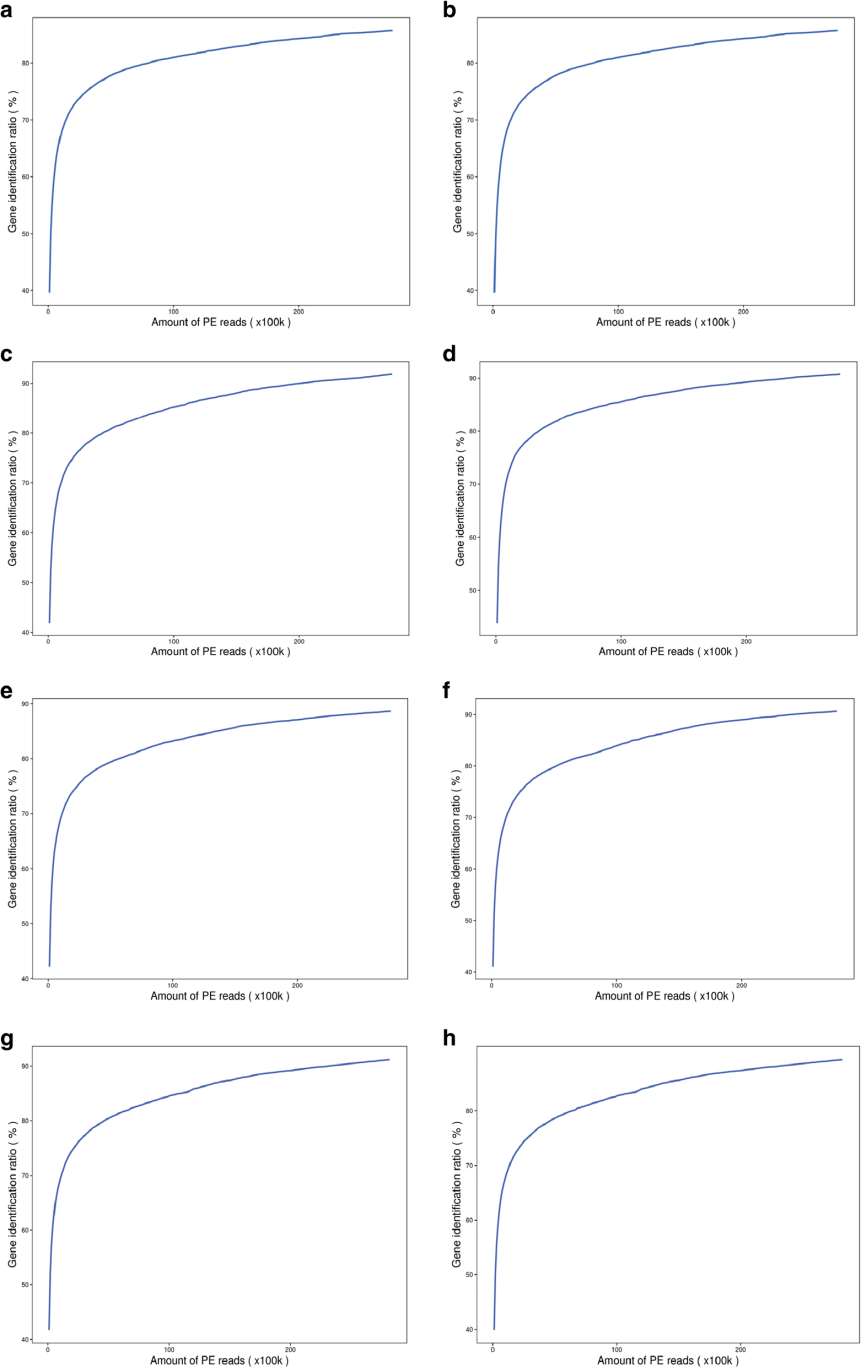
Analysis of sequencing saturation. **a** HRK0–1, **b**HRK0–2, **c** HRK48–1, **d** HRK48–2, **e** HSK0–1, **f** HSK0–2, **g** HSK48–1, **h** HSK48–2

An Illumina HiSeq™2000 sequencer was employed to analyze the comparative transcriptome of eight samples of Gantai-2-2 and Wan82–178 leaves that bean pyralid had been feeding on 0 h and 48 h. The original image data obtained by sequencing base-calling were the original sequence reads. Each read in the Solexa paired-end (PE) sequencing was 100 bp in length. There were 45.88 G original data sets produced during sequencing. The mean sequencing depth was 5.67. After the raw data were trimmed, 442,422,398 clean reads were obtained. The clean/raw read rates of the eight samples ranged from 95.30% to 97.11%. All clean reads were matched to the soybean reference genome by BWA software, allowing two base mismatches. The mapped genome reads ranged from 42,474,863 to 44,489,050 sets, genome map rates ranged from 77.23% to 78.95%, and unique match rates ranged from 72.86% to 75.22%. The expressed genes ranged from 50,283 to 53,739 (Table 1). To estimate whether the sequencing depth was sufficient for transcriptome coverage, the sequencing saturation in the eight cDNA libraries was analyzed. The results showed that most genes became saturated when the amount of PE reads was 20 M (200 × 100 kb) (Fig. 1), which indicated that the overall quality of sequencing saturation in the eight cDNA libraries was high and that the sequencing *amount* covered the vast majority of expressed genes.

**Table 1:** Number of reads sequenced and mapped to soybean genome

	HRK0–1	HRK0–2	HRK48–1	HRK48–2	HSK0–1	HSK0–2	HSK48–1	HSK48–2	Sum
Raw reads	56,776,842	56,776,934	56,776,686	56,777,138	56,777,168	56,777,236	59,048,232	59,048,102	458,758,338
Clean reads	54,999,276	55,134,342	54,619,378	54,911,596	54,809,878	55,085,486	56,273,358	56,589,084	442,422,398
≥Q20(%)	97.22	97.45	97.51	97.55	97.17	97.57	97.69	97.68	–
Clean reads/Raw reads (%)	96.87	97.11	96.20	96.71	96.54	97.02	95.30	95.84	–
Genome map rates (%)	77.23	78.07	78.25	78.28	78.32	78.95	78.52	78.62	–
Unique Match (%)	72.86	74.01	74.94	75.22	74.50	74.46	74.76	74.89	–
Expressed gene	50,283	51,399	51,935	53,739	51,469	51,794	51,835	53,032	70,016

*HRK* represented the highly resistant line Gantai-2-2; *HSK* represented the highly susceptible line Wan82–178; numbers 0 and 48 represented the processing time; and −1 and −2 represented repetitions 1 and 2, respectively. Sequence length was 2 × 100 bp, length of each read was 100 bp using double end sequencing

### Correlation analysis of samples

**Fig. 2:**
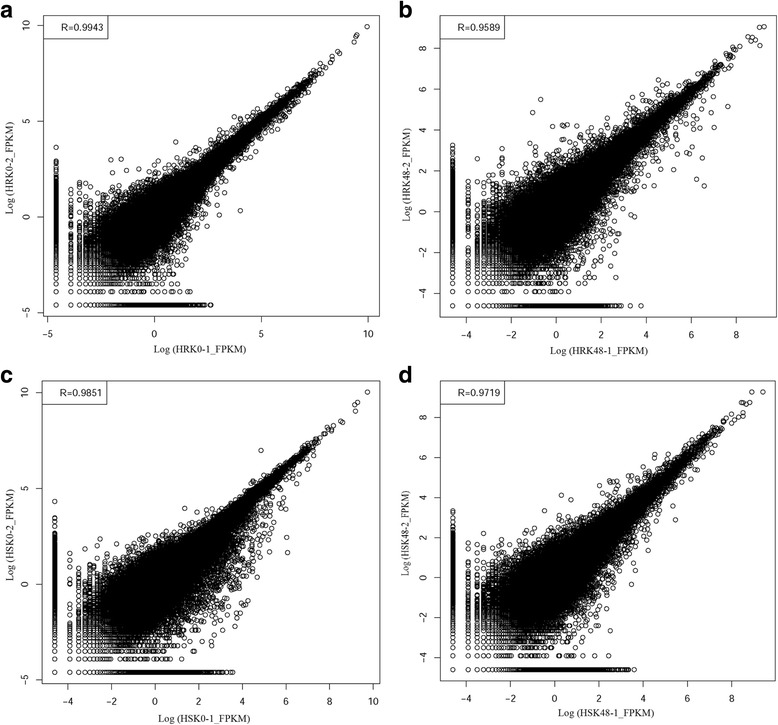
Correlations value of each repetition. **a** HRK0–1 and HRK0–2. **b** HRK48–1 and HRK48–2. **c** HSK0–1 and HSK0–2. **d** HSK48–1 and HSK48–2

The correlation of gene expression levels among samples is a key criterion to test whether the experiments are reliable and whether the chosen samples are reasonable. If one sample is highly similar to another one, the correlation value between them is very close to 1. We calculated the correlation value between each of two samples based on the FPKM results. According to the standard that Encode plan recommends, the square of the correlation value (R^2^) should be ≥0.92 (under an ideal experimental environment with reasonable samples). Our results showed that the R^2^ of all repetitions were >0.95 (Fig. 2), which signified that our experimental samples and results were satisfactory and reliable.

### Screening of differentially expressed genes (DEGs)

**Fig. 3:**
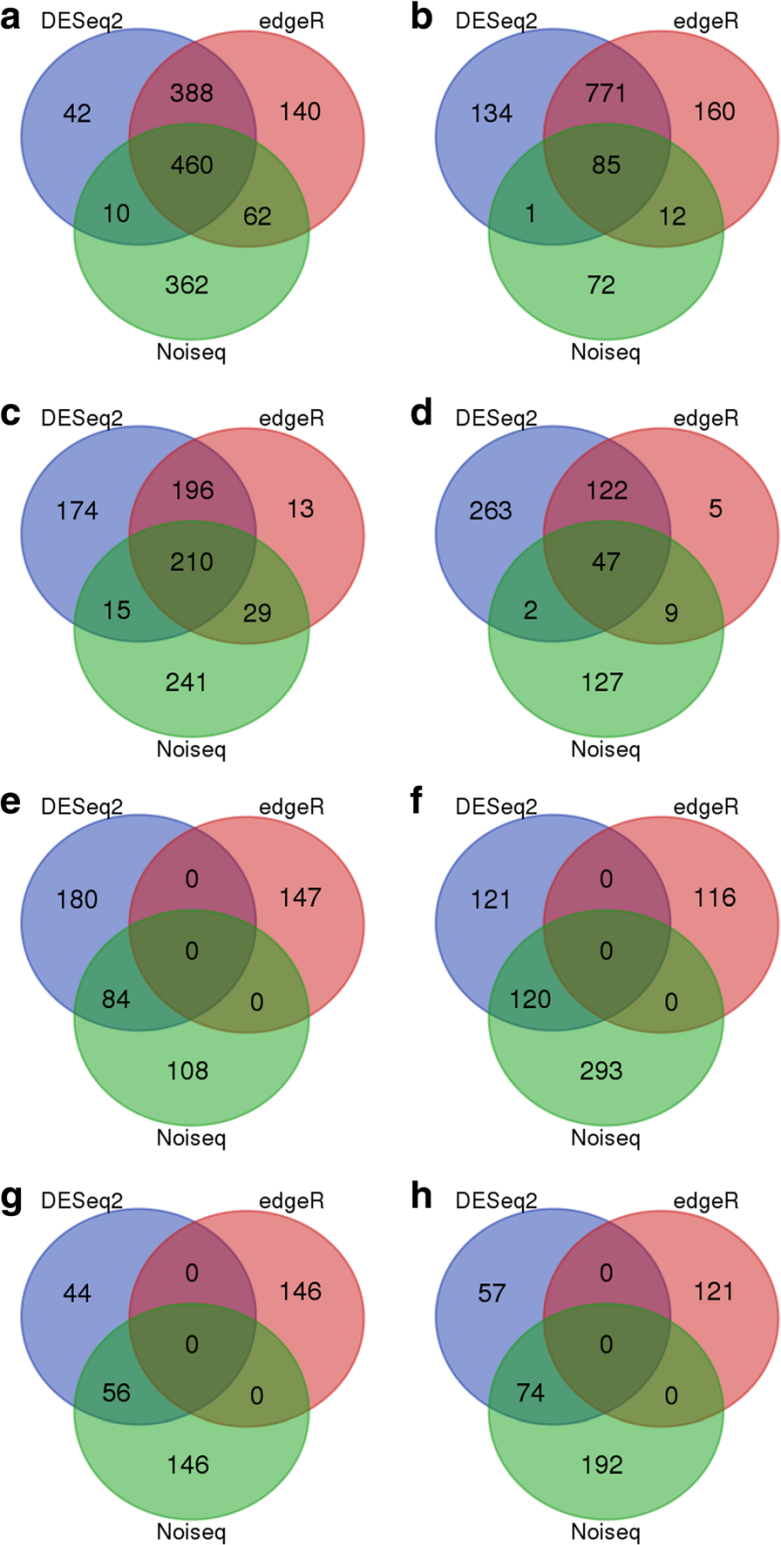
The DEGs were screened by Noiseq, DESeq2 and edgeR. **a** HRK48/HRK0_UP In total, 894, 900 and 1050 up-regulated DEGs were identified by Noiseq, DESeq2 and edgeR, respectively. 460 DEGs were identified under the three methods, 62 DEGs were identified under both Noiseq and edgeR, 388 DEGs were identified under both DESeq2 and edgeR, 10 DEGs were identified under both Noiseq and DESeq2. **b** HRK48/HRK0_DOWN In total, 170, 991 and 1028 down-regulated DEGs were identified by Noiseq, DESeq2 and edgeR, respectively. 85 DEGs were identified under the three methods, 12 DEGs were identified under both Noiseq and edgeR, 771 DEGs were identified under both DESeq2 and edgeR, 1 DEGs were identified under both Noiseq and DESeq2. **c** HSK48/HSK0_UP In total, 495, 595 and 448 up-regulated DEGs were identified by Noiseq, DESeq2 and edgeR, respectively. 210 DEGs were identified under the three methods, 29 DEGs were identified under both Noiseq and edgeR, 196 DEGs were identified under both DESeq2 and edgeR, 15 DEGs were identified under both Noiseq and DESeq2. **d** HSK48/HSK0_DOWN In total, 185, 434 and 183 down-regulated DEGs were identified by Noiseq, DESeq2 and edgeR, respectively. 47 DEGs were identified under the three methods, 9 DEGs were identified under both Noiseq and edgeR, 122 DEGs were identified under both DESeq2 and edgeR, 2 DEGs were identified under both Noiseq and DESeq2. **e** HRK0/HSK0_UP In total, 192, 264 and 147 up-regulated DEGs were identified by Noiseq, DESeq2 and edgeR, respectively. 84 DEGs were identified under both Noiseq and DESeq2. **f** HRK0/HSK0_DOWN In total, 413, 241 and 116 down-regulated DEGs were identified by Noiseq, DESeq2 and edgeR, respectively. 120 DEGs were identified under both Noiseq and DESeq2. **g** HRK48/HSK48_UP In total, 202, 100 and 146 up-regulated DEGs were identified by Noiseq, DESeq2 and edgeR, respectively. 56 DEGs were identified under both Noiseq and DESeq2. **h** HRK48/HSK48_DOWN In total, 266, 131 and 121 down-regulated DEGs were identified by Noiseq, DESeq2 and edgeR, respectively. 74 DEGs were identified under both Noiseq and DESeq2

Noiseq, DESeq2 and edgeR methods were used to screen DEGs (Fig. 3). The results showed that the Noiseq method can screen DEGs between two groups, showing a good performance when comparing it to other differential expression methods, such as edgeR and DESeq2. Noiseq maintains good True Positive and False Positive rates when the sequencing depth is increased, whereas most other methods show poor performance. Further more, Noiseq models the noise distribution from the actual data to better adapt to the size of the data set and be more effective in controlling the rate of false discoveries. Therefore, the Noiseq method was used to screen the DEGs.

As a result, under bean pyralid larvae feeding for 48 h, 1064 DEGs were identified in the Gantai-2-2, of which 894 DEGs were up-regulated and 170 DEGs were down-regulated compared to 0 h (Additional file 1: Table S1). Additionally, 680 DEGs were identified in Wan82–178, of which 495 DEGs were up-regulated and 185 DEGs were down-regulated (Additional file 2: Table S2). After being induced with bean pyralid larvae, the number of up-regulated genes was significantly higher than that of down-regulated genes. These results indicated that most of the genes were activate and a few genes were inhibited after insect feeding.

To screen the constitutive defense genes, the highly resistant line and highly sensitive line were compared at different feeding times. The results showed that 605 DEGs were identified in Gantai-2-2 at 0 h of feeding, of which 192 DEGs were up-regulated and 413 DEGs were down-regulated (Additional file 3: Table S3), compared to Wan82–178. And at 48 h feeding, 468 DEGs were identified in Gantai-2-2, of which 202 DEGs were up-regulated and 266 DEGs were down-regulated (Additional file 4: Table S4), compared to Wan82–178.

**Fig. 4:**
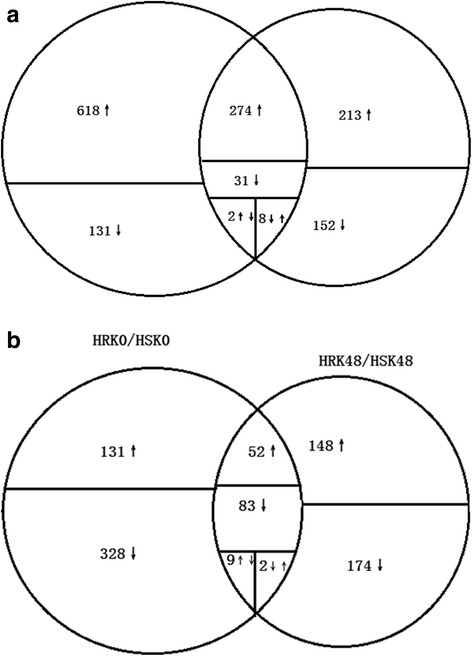
Venn diagram of the distribution of DEGs. **a** HRK48/HRK0 and HSK48/HSK0. **b** HRK0/HSK0 and HRK48/HSK48. The circles are proportional to the number of genes identified in each treatment. The overlapping regions indicate the number of common genes. The ↑ indicate up-regulated, ↓ indicate down-regulated, ↑↓ indicate up-regulated in HRK48/HRK0 or HRK0/HSK0 but down-regulated in HSK48/HSK0 or HRK48/HSK48, ↓↑ indicate up-regulated in HSK48/HSK0 or HRK48/HSK48 but down-regulated in HRK48/HRK0 or HRK0/HSK0

The DEGs were further divided into three categories. The first category was the “DEGs with non-bean pyralid-induced genotype”, and there were 605 DEGs in total. This class of genes was the “DEGs identified in Gantai-2-2 compared to Wan 82-178 before bean pyralid feeding induction”, in which 52 DEGs were always up-regulated and 83 DEGs were always down-regulated at 0 h and 48 h. In addition, 9 DEGs were up-regulated at 0 h but down-regulated at 48 h, and 2 DEGs were down-regulated at 0 h but up-regulated at 48 h, whereas the other 459 DEGs displayed no changes at 48 h (Fig. 4a). The second category was the “bean pyralid-induced DEGs that appeared in both materials”. This category included 315 DEGs, which mainly displayed an up-regulated trend, with 274 DEGs were up-regulated and 31 DEGs were down-regulated in the two materials. A total of 8 DEGs were down-regulated in Gantai-2-2 but up-regulated in Wan82–178, 2 DEGs up-regulated in Gantai-2-2 but down-regulated in Wan82–178 (Fig. 4b). The third type was the “bean pyralid-induced genotype DEGs”, which consisted of a total of 1114 DEGs, of which 749 DEGs were only expressed in Gantai-2-2 and 365 DEGs were only expressed in Wan82–178.

### Gene Ontology (GO) annotation analysis of the DEGs

**Fig. 5:**
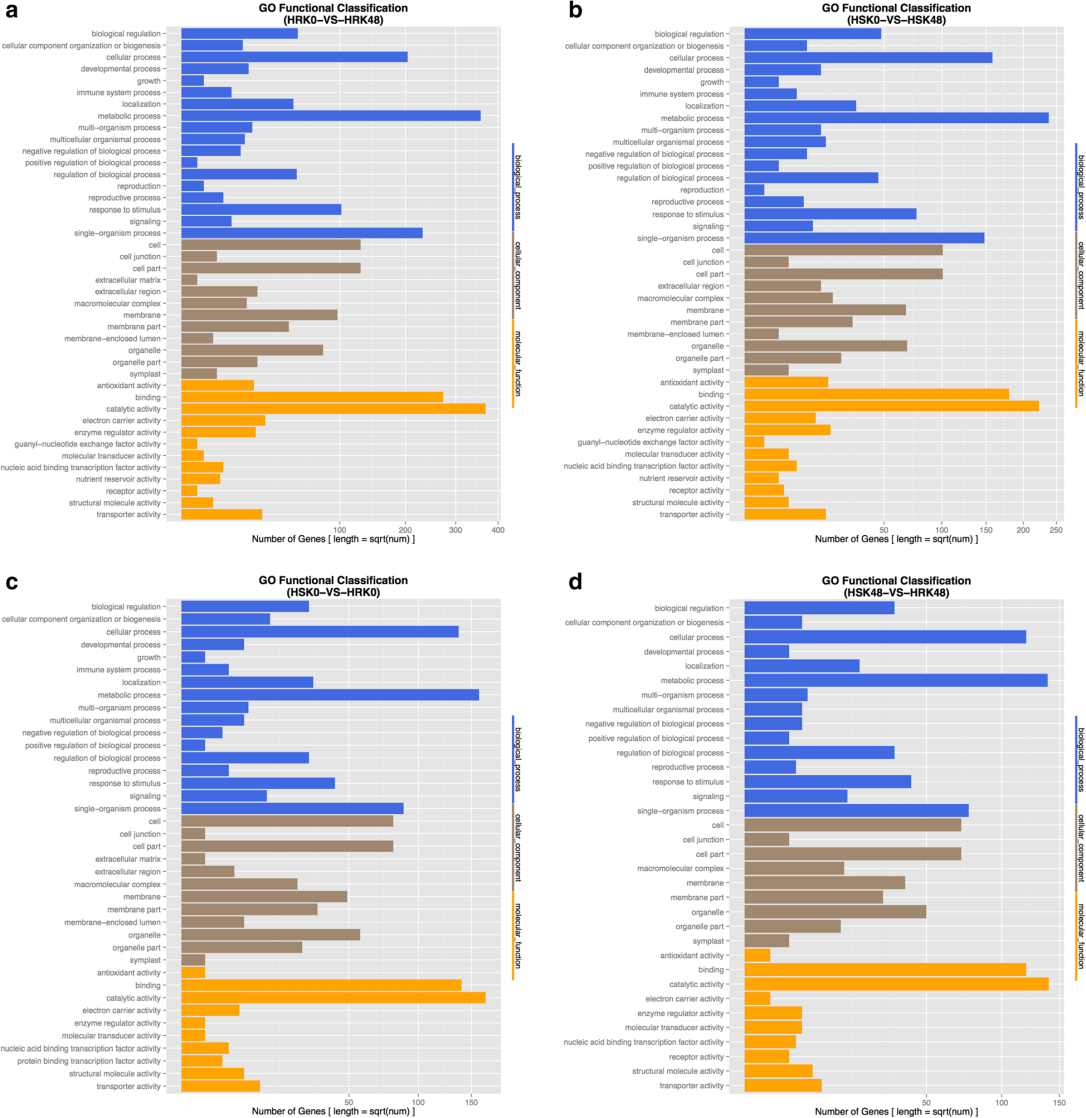
GO function analysis of the DEGs. **a** HRK48/HRK0. **b** HSK48/HSK0. **c** HRK0/HSK0. **d** HRK48/HSK48

To further analyze the cellular components, molecular functions and biological processes of the DEGs, GO annotation analysis was performed on all of the identified DEGs. The results showed that, under bean pyralid larvae feeding for 48 h, 572 DEGs (53.76%) of Gantai-2-2 were annotated to 42 functional groups, including 18 biological processes, 12 cellular components and 12 molecular functions compared to 0 h (Fig. 5a, Additional file 5: Table S5). Under bean pyralid larvae feeding for 48 h, 378 DEGs (55.59%) of Wan82–178 were annotated to 41 functional groups, including 18 biological processes, 11 cellular components and 12 molecular functions compared to 0 h (Fig. 5b, Additional file 6: Table S6). When comparing Gantai-2-2 with Wan82–178 at 0 h feeding, 285 DEGs (47.11%) were annotated to 39 functional groups, including 17 biological processes, 12 cellular components and 10 molecular functions (Fig. 5c, Additional file 7: Table S7). When comparing Gantai-2-2 with Wan82–178 at 48 h feeding, 240 DEGs (51.28%) were annotated to 34 functional groups, including 15 biological processes, 9 cellular components and 10 molecular functions (Fig. 5d, Additional file 8: Table S8).

Among GO annotations of the DEGs in the above four groups, the largest common functional groups were metabolic process, cellular process, single-organism process, responses to stimuli, catalytic activities and binding. These results indicated that when the soybean was subjected to bean pyralid larvae feeding, the defense systems in the plants would immediately respond to the stimuli, appropriately increase metabolic activities in vivo and produce defense substances, such as defendant enzymes, and proteinase inhibitors, thereby enhancing the activities of various enzymes to promote defense.

### Pathway analysis of the DEGs

**Fig. 6:**
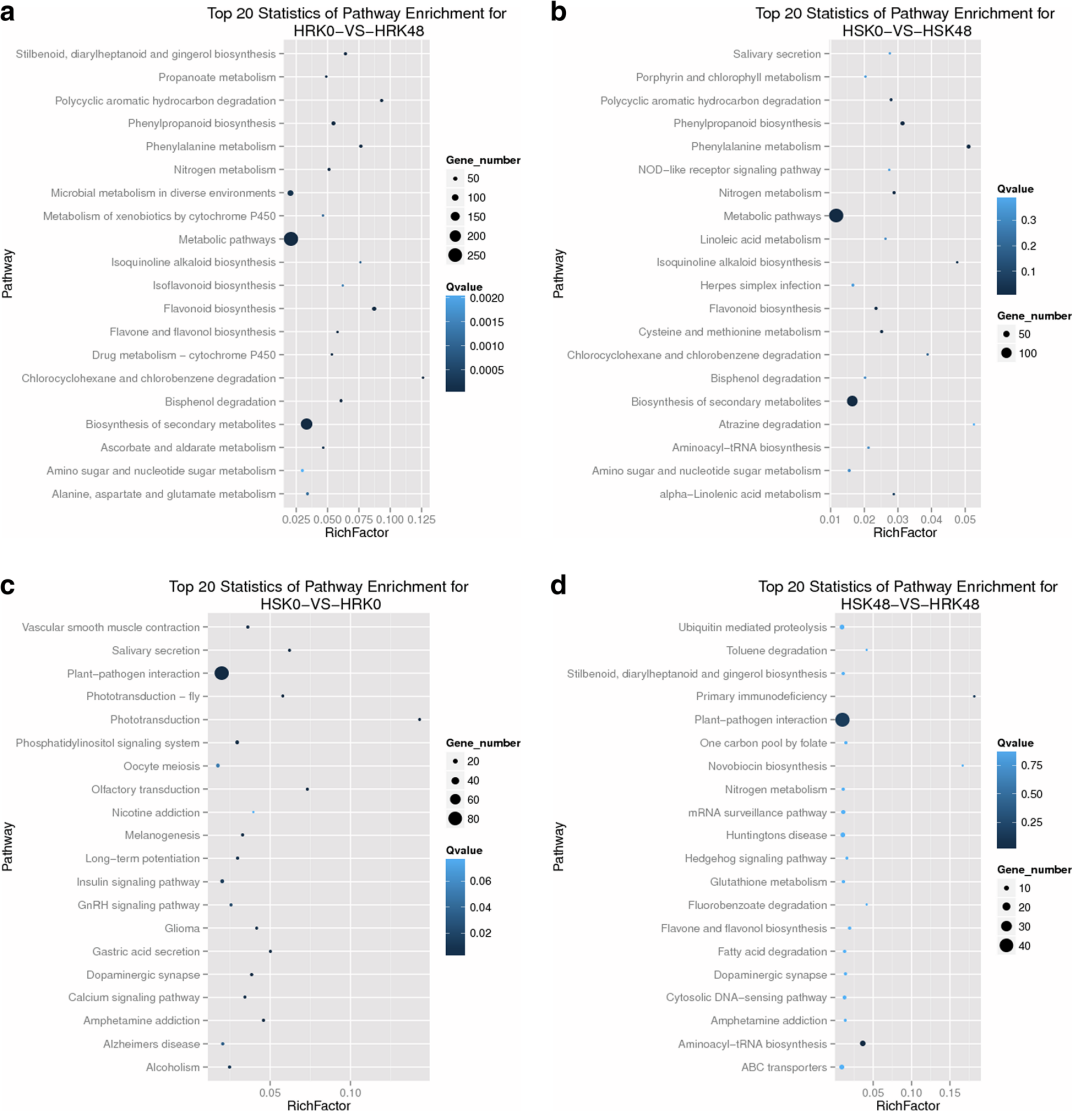
Top 20 pathway entries of the DEGs.**a** HRK48/HRK0. **b** HSK48/HSK0. **c** HRK0/HSK0. **d** HRK48/HSK48

Pathway-based analysis was performed using the Kyoto Encyclopedia of Genes and Genomes (KEGG) pathway database. The results showed that 614 DEGs (57.71%) of Gantai-2-2 were assigned to 256 pathways under bean pyralid larvae feeding for 48 h, compared to 0 h. Which mainly included the metabolic pathways (263, 42.83%), biosynthesis of secondary metabolites (206, 33.55%), microbial metabolism in a diverse environment (85, 13.84%), flavonoid biosynthesis (52, 8.47%), phenylpropanoid biosynthesis (47, 7.65%) and phenylalanine metabolism (33, 5.37%) (Additional file 9: Table S9, Fig. 6a). And 380 DEGs (55.88%) of Wan82–178 were assigned to 224 pathways under bean pyralid larvae feeding for 48 h, compared to 0 h, which mainly included the metabolic pathways (147, 38.68%), biosynthesis of secondary metabolites (102, 26.84%), phenylpropanoid biosynthesis (27, 7.11%), phenylalanine metabolism (22, 5.79%) and flavonoid biosynthesis (14, 3.68%) (Additional file 10: Table S10, Fig. 6b). When comparing Gantai-2-2 with Wan82–178 at 0 h feeding, 367 DEGs (62.15%) were assigned to 208 pathways, which mainly included plant-pathogen interaction (84, 22.89%), insulin signaling pathway (14, 3.81%), phosphatidylinositol signaling system (13, 3.54%), and oocyte meiosis (13, 3.54%) (Additional file 11: Table S11, Fig. 6c). When comparing Gantai-2-2 with Wan82–178 at 48 h feeding, 209 DEGs (63.68%) were assigned to 209 pathways, which mainly included plant-pathogen interaction (42, 14.09%), aminoacyl-tRNA biosynthesis (12, 4.03%), ABC transporters (10, 3.36%) and huntington’s disease (9, 3.02%) (Additional file 12: Table S12, Fig. 6d).

Pathway analysis showed that differentially expressed of these genes in the metabolic pathways after induced by bean pyralid might be related to the resistance, such as plant-pathogen interactions, phenylpropanoid biosynthesis, phenylalanine metabolism, flavonoid biosynthesis, peroxisome, plant hormone signal transduction, terpenoid backbone biosynthesis, and so on, and that they played a defensive role against insect stress.

**Fig. 7:**
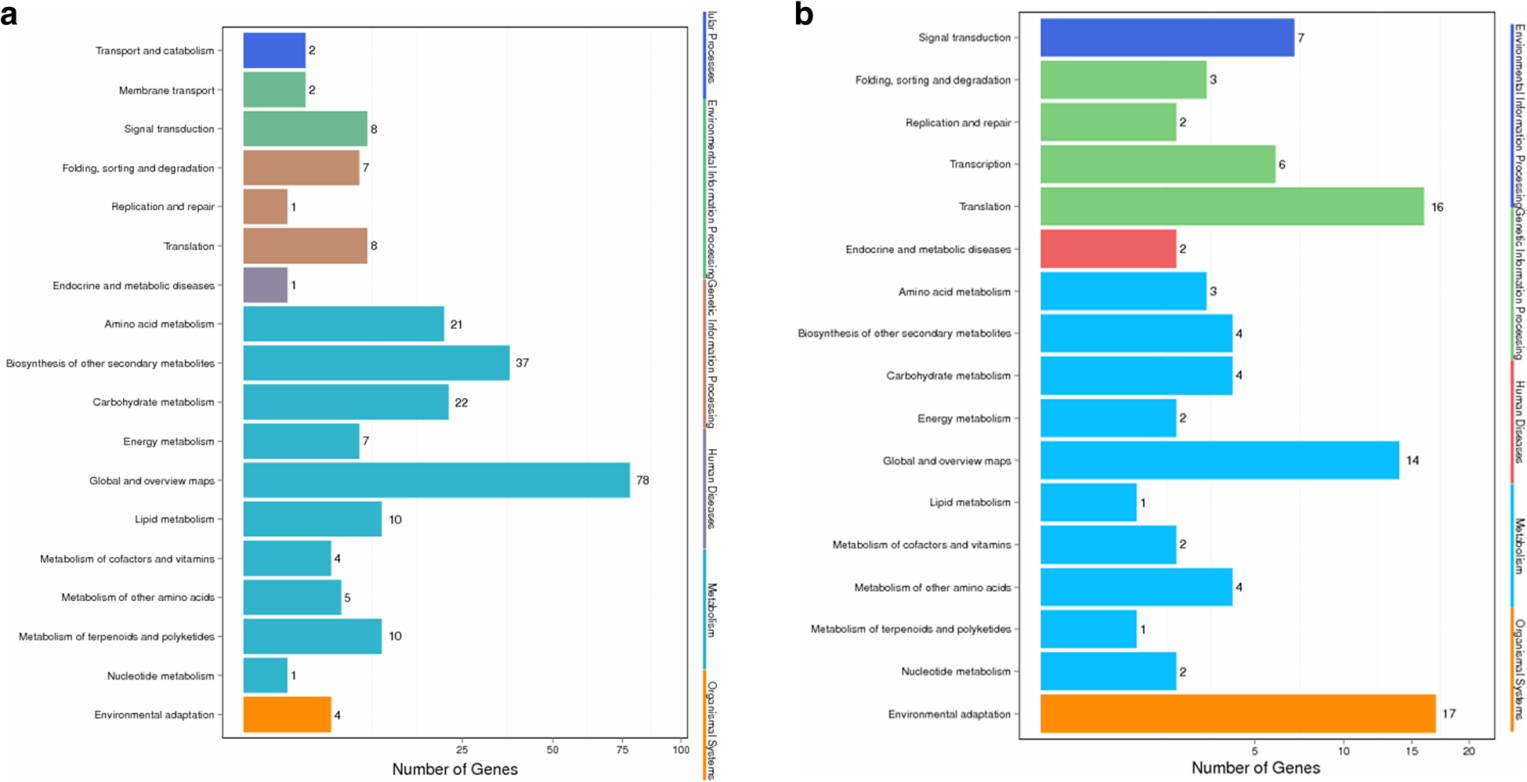
Pathway classification of the DEGs. a Pathway classification of “bean pyralid-induced DEGs which appeared in both materials”. **b** DEGs were identified in Gantai-2-2 compared to Wan 82–178 before and after bean pyralid feeding Note: The X axis represent the percent of genes (%), and the Y axis represent the metabolic process

R language was used to conduct a super geometric algorithm. The result showed that the DEGs belonged to the bean pyralid-induced DEGs that appeared in both materials, mainly involving the global and overview maps, biosynthesis of the other secondary metabolites, carbohydrate metabolism and amino acid metabolism (Fig. 7a). A total of 146 DEGs were identified in Gantai-2-2 compared to Wan 82–178 before and after bean pyralid feeding that were mainly involved environmental adaptation, translation, global and overview maps and signal transduction (Fig. 7b).

### Analysis of DEGs potentially related to anti-bean pyralid in soybean

Man pathway cluster analysis was employed to identify the pathway classification of the DEGs. The results showed that some important DEGs were categorized into ROS removal, hormone metabolism, signaling, stress, secondary metabolism and cell wall (Tables 2 and 3).

**Table 2:** Functional classification of DEGs

Functional category	Pathways	HRK48/HRK0		HSK48/HSK0		HRK0/HSK0		HRK48/HSK48	
		up	down	up	down	up	down	up	down
ROS removal	Peroxidases	23	0	20	0	0	0	0	0
	Polyphenol oxidase	5	0	4	0	0	0	0	0
	Glutathione S transferases	9	0	2	0	0	1	0	0
	Thioredoxin 1	3	0	0	0	0	0	1	0
Hormone metabolism	Jasmonate	8	0	6	0	0	2	0	2
	Ethylene	22	2	14	0	1	0	1	1
	Abscisic acid	1	0	2	0	0	2	0	1
	Auxin	12	0	6	0	1	0	1	0
	Brassinosteroid	1	0	0	0	0	0	0	0
	Cytokinin	0	2	1	0	0	0	0	0
	Gibberelin	3	0	1	0	0	0	0	0
	Salicylic acid	0	0	1	0	0	0	0	1
Signalling	Protein kinases	17	0	6	1	7	26	8	7
	Calcium	6	1	3	5	4	15	4	1
Stress	Biotic	17	2	8	0	1	3	2	1
	Biotic .signalling	1	0	0	0	0	0	0	0
	Biotic.PR-proteins	10	2	5	2	1	12	3	6
	Biotic. proteinase inhibitors	8	0	7	0	0	0	3	0
	Abiotic	2	0	1	0	0	0	0	0
	Abiotic.heat	6	0	4	2	0	6	0	2
	Abiotic.cold	1	0	0	1	0	1	2	0
	Abiotic.drought/salt	1	2	1	1	2	1	0	0
	Abiotic.touch/wounding	1	0	1	0	0	0	0	0
	Abiotic.unspecified	9	4	5	2	1	1	1	1
Secondary metabolism	Isoprenoid	6	0	6	0	0	1	0	1
	Phenylpropanoid	13	0	4	0	2	4	2	3
	Flavonoid	22	0	4	0	0	3	1	1
	Cytochrome P450	10	1	2	0	1	1	1	0
Cell wall	Simple phenol modification	4 7	0 3	1 2	0 2	0 0	0 0	0 3	0 0

*HRK* represented the highly resistant line Gantai-2-2; *HSK* represented the highly susceptible line Wan82–178; and the numbers 0 and 48 represented the processing times

**Table 3:** Comparison of some DEGs of the Gantai-2-2 and Wan82–178 after bean pyralid larvae feeding

Gene ID	Gene Annotation	HRK48/HRK0	HSK48/HSK0	HRK0/HSK0	HRK48/HSK48
*Peroxidases*					
Glyma.20G169200.1	Peroxidases	4.34	3.79	–	–
Glyma.04G220600.1	Peroxidases	5.72	5.72	–	–
Glyma.10G222400.1	Peroxidases	4.36	4.78	–	–
Glyma.15G128800.1	Peroxidases	5.53	5.42	–	–
Glyma.09G277900.1	Peroxidases	6.46	5.68	–	–
Glyma.06G145300.1	Peroxidases	4.59	4.82	–	–
Glyma.09G277800.1	Peroxidases	5.42	4.19	–	–
Glyma.10G050800.1	Peroxidases	9.64	7.48	–	–
Glyma.06G275900.1	Peroxidases	9.64	5.10	–	–
Glyma.02G233800.1	Peroxidases	7.92	8.11	–	–
Glyma.09G022400.1	Peroxidases	4.81	6.44	–	–
Glyma.18G211000.1	Peroxidases	5.19	4.34	–	–
Glyma.08G179700.1	Peroxidases	5.46	4.91	–	–
Glyma.13G138300.1	Peroxidases	8.10	6.22	–	–
Glyma.18G211100.1	Peroxidases	6.68	5.39	–	–
Glyma.16G164400.1	Peroxidases	8.58	10.14	–	–
Glyma.12G129500.1	Peroxidases	4.64	5.70	–	–
Glyma.15G128700.1	Peroxidases	5.37	3.92	–	–
Glyma.08G179600.1	Peroxidases	3.47	3.76	–	–
Glyma.16G164200.1	Peroxidases	9.65	6.46	–	–
Glyma.15G052700.1	Peroxidases	6.73	–	–	–
Glyma.11G162100.1	Peroxidases	7.75	–	–	–
Glyma.09G022300.1	Peroxidases	5.92	–	–	–
*Polyphenol oxidase*					
Glyma.04G121700.1	Polyphenol oxidase	6.95	–	–	–
Glyma.07G193400.1	Polyphenol oxidase	7.54	–	–	–
Glyma.06G270400.1	Polyphenol oxidase	7.87	7.11	–	–
Glyma.15G071200.1	Polyphenol oxidase	9.17	8.94	–	–
Glyma.13G242300.1	Polyphenol oxidase	7.14	–	–	–
*Glutathione S-transferase*					
Glyma.11G198500.1	Glutathione S-transferase	6.40	6.15	–	–
Glyma.20G020300.4	Putative glutathione S-transferase	7.48	9.37	–	–
Glyma.13G129000.1	Glutathione S-transferase	3.63	–	–	–
Glyma.02G024800.1	Glutathione S-transferase	8.21	–	–	–
Glyma.10G192900.1	Glutathione S-transferase	6.52	–	–	–
Glyma.08G118700.1	Glutathione S-transferase	4.93	–	–	–
Glyma.02G024600.1	Glutathione S-transferase	9.35	–	–	–
Glyma.07G139800.1	Glutathione S-transferase	4.52	–	–	–
Glyma.07G139700.1	Glutathione S-transferase	5.19	–	−4.77	–
*Thioredoxin*					
Glyma.12G215000.1	Thioredoxin 1	5.87	–	–	–
Glyma.18G255200.3	Thioredoxin 1	8.15	–	–	–
Glyma.06G266700.1	Thioredoxin 1	7.91	–	–	7.91
*Hormone metabolism*					
Glyma.07G034900.1	Linoleate 9S–lipoxygenase	5.23	6.54	–	–
Glyma.15G026400.2	Linoleate 9S–lipoxygenase	4.13	3.75	–	–
Glyma.07G034800.1	Linoleate 9S–lipoxygenase	4.73	–	−4.64	–
Glyma.13G030300.1	Lipoxygenase	5.90	3.07	–	–
Glyma.13G030300.2	Lipoxygenase	–	–	–	−8.93
Glyma.04G035000.1	Hydroperoxide dehydratase	5.99	5.74	–	–
Glyma.13G109800.1	12-Oxophytodienoic acid reductase	7.41	7.82	–	–
Glyma.15G223900.1	12-Oxophytodienoic acid reductase	9.26	–	–	–
Glyma.176209900.1	12-Oxophytodienoic acid reductase	–	–	−6.70	−10.45
Glyma.19G011700.1	Alpha-dioxygenas	4.43	3.15	–	–
Glyma.17G178300.2	Aminocyclopropanecarboxylate oxidase	7.02	8.71	–	–
Glyma.01G056100.1	Aminocyclopropanecarboxylate oxidase	7.73	6.08	–	–
Glyma.08G092800.1	Aminocyclopropanecarboxylate oxidase	8.18	5.71	–	–
Glyma.09G107100.1	Aminocyclopropanecarboxylate oxidase	4.64	4.79	–	–
Glyma.16G017500.1	Aminocyclopropanecarboxylate oxidase	6.79	5.11	–	–
Glyma.02G268200.1	Aminocyclopropanecarboxylate oxidase	9.18	–	–	–
Glyma.02G268000.4	Aminocyclopropanecarboxylate oxidase	9.49	–	–	–
Glyma.02G268000.3	Aminocyclopropanecarboxylate oxidase	–	–	3.66	–
Glyma.09G002600.15	Ethylene receptor	7.47	–	–	–
Glyma.09G002600.12	Ethylene receptor	–	9.06	–	–
Glyma.09G002600.14	Ethylene receptor	–	–	–	4.98
Glyma.03G251700.3	Ethylene receptor	–	–	–	−7.07
Glyma.10G007000.1	Ethylene-responsive transcription factor 1	6.46	–	–	–
Glyma.10G186800.1	Ethylene-responsive transcription factor 1	5.98	–	–	–
Glyma.20G070100.1	EREBP-like factor	3.48	3.42	–	–
Glyma.13G279200.1	IAA-amino acid hydrolase	3.38	3.03	–	–
Glyma.13G352400.2	IAA-amino acid hydrolase	8.65	–	–	–
Glyma.15G022300.1	IAA-amino acid hydrolase	3.83	–	–	–
Glyma.06G115100.1	IAA-amino acid hydrolase	3.21	–	–	–
Glyma.08G010400.1	SAUR family protein	9.27	5.23	–	–
Glyma.06G006500.1	SAUR family protein	8.60	9.21	–	–
Glyma.12G150500.1	SAUR family protein	6.19	–	–	–
Glyma.04G006600.1	SAUR family protein	–	10.13	–	–
Glyma.06G282000.1	SAUR family protein	–	–	–	5.33
Glyma.12G222400.1	Asparagine synthase (glutamine-hydrolysing)	3.87	3.85	–	–
Glyma.12G150500.1	Asparagine synthase (glutamine-hydrolysing)	3.87	7.34	–	–
Glyma.15G072400.1	Asparagine synthase (glutamine-hydrolysing)	3.74	–	–	–
Glyma.15G071300.3	Asparagine synthase (glutamine-hydrolysing)	9.47	–	–	–
Glyma.01G190600.1	Auxin responsive GH3 gene family	6.83	–	–	–
Glyma.U019800.1	ARF	−7.57	–	–	–
*Protein kinases*					
Glyma.05G220200.4	Protein kinase	8.92	–	–	–
Glyma.14G116000.7	Protein kinase	7.77	–	–	–
Glyma.07G253900.1	Protein kinase A	7.99	–	–	–
Glyma.17G029200.1	Protein kinase A	–	8.43	–	–
Glyma.18G242700.2	Protein kinase A	–	–	−7.71	–
Glyma.17G029200.1	Protein kinase A	–	–	–	−8.43
Glyma.15G209300.1	LRR receptor-like serine/threonine-protein kinase FLS2	8.52	8.32	–	–
Glyma.08G128900.1	LRR receptor-like serine/threonine-protein kinase FLS2	5.16	–	–	–
Glyma.06G319700.1	LRR receptor-like serine/threonine-protein kinase FLS2	5.14	–	–	–
Glyma.19G145200.1	LRR receptor-like serine/threonine-protein kinase FLS2	7.35	–	–	–
Glyma.17G250800.2	LRR receptor-like serine/threonine-protein kinase FLS2	9.25	–	–	–
Glyma.08G079100.1	LRR receptor-like serine/threonine-protein kinase FLS2	–	6.36	–	–
Glyma.16G185100.1	LRR receptor-like serine/threonine-protein kinase FLS2	–	–	–	6.59
Glyma.01G004800.1	Serine/threonine-protein kinase PBS1(STK)	7.57	–	–	–
Glyma.09G272300.7	Serine/threonine-protein kinase PBS1(STK)	7.76	–	–	–
Glyma.18G217000.4	Serine/threonine-protein kinase PBS1(STK)	8.11	–	–	–
Glyma.09G063200.2	Serine/threonine-protein kinase PBS1(STK)	–	8.13	–	–
Glyma.13G216100.1	Serine/threonine-protein kinase PBS1(STK)	–	8.12	9.54	–
Glyma.06G081800.1	Serine/threonine-protein kinase PBS1(STK)	–	−7.67	–	–
Glyma.20G137300.1	Serine/threonine-protein kinase PBS1(STK)	–	–	9.15	–
Glyma.17G039800.2	Serine/threonine-protein kinase PBS1(STK)	–	–	8.75	–
Glyma.16G185100.1	Serine/threonine-protein kinase PBS1(STK)	–	–	5.92	–
Glyma.20G137300.1	Serine/threonine-protein kinase PBS1(STK)	–	–	–	9.76
Glyma.17G039800.2	Serine/threonine-protein kinase PBS1(STK)	–	–	–	8.60
Glyma.19G036600.2	Serine/threonine-protein kinase PBS1(STK)	–		–	7.78
Glyma.05G066700.1	Serine/threonine-protein kinase SRK2	7.86	–	–	–
Glyma.01G204200.4	Serine/threonine-protein kinase SRK2	8.39	–	–	–
Glyma.18G054100.1	Serine/threonine-protein kinase WNK1	4.26	3.80	–	–
Glyma.10G092400.1	Serine/threonine-protein kinase WNK1	–	–	7.69	–
Glyma.20G105300.5	Serine/threonine-protein kinase CTR1	8.33	–	–	–
Glyma.07G197200.2	Serine/threonine-protein kinase CTR1	5.89	–	–	–
Glyma.20G105300.1	Serine/threonine-protein kinase CTR1	7.87	–	–	–
Glyma.03G232400.2	Calmodulin	3.52	–	–	–
Glyma.05G237200.1	Calmodulin	–	−3.34	–	–
Glyma.09G182400.1	Calmodulin	–	−4.57	–	–
Glyma.05G028600.4	Calmodulin	–	–	8.98	–
Glyma.13G271800.4	Calmodulin	–	–	8.05	–
Glyma.08G127700.2	Calmodulin	–	–	7.71	–
Glyma.03G178200.2	Calmodulin	–	–	–	9.28
Glyma.08G044400.2	Calmodulin	–	–	−3.37	–
Glyma.07G093900.1	Calmodulin	–	–	−5.80	–
Glyma.03G232500.2	Calmodulin	–	–	−4.66	–
Glyma.12G103600.1	Calmodulin	–	–	−4.82	–
Glyma.19G229400.1	Calmodulin	–	–	−4.67	–
Glyma.09G182400.1	Calmodulin	–	–	−5.32	–
Glyma.05G237200.1	Calmodulin	–	–	−4.32	–
Glyma.03G232400.2	Calmodulin	–	–	−4.67	–
Glyma.11G147500.4	Calcium/calmodulin-dependent protein kinase	–	7.67	–	–
Glyma.18G096500.3	Calcium/calmodulin-dependent protein kinase	–	–	−7.61	–
Glyma.14G068400.1	Calcium/calmodulin-dependent protein kinase	–	–	−4.06	–
Glyma.06G098900.2	Calcium/calmodulin-dependent protein kinase	–	–	–	7.96
Glyma.03G246800.1	Calcium-binding protein CML	4.35	–	–	–
Glyma.16G095700.5	Calcium-binding protein CML	8.09	–	–	–
Glyma.06G034700.1	Calcium-binding protein CML	–	−3.39	−4.34	–
Glyma.20G034200.1	Calcium-binding protein CML	–	−7.80	–	–
Glyma.08G265200.2	Calcium-binding protein CML	–	9.50	8.17	8.01
Glyma.02G207800.2	Calcium-binding protein CML	–	–	−8.41	
Glyma.02G186900.1	Calcium-binding protein CML	–	–	8.50	8.49
Glyma.16G214500.1	Calcium-binding protein CML	–	–	−4.78	−5.30
Glyma.18G260700.1	Calcium-binding protein CML	–	–	−3.50	–
Glyma.02G108700.1	Calcium-binding protein CML	–	–	−4.88	–
Glyma.16G059300.1	Calcium-binding protein CML	–	–	−6.04	–
Glyma.07G004300.2	Ca^2+^-transporting ATPase	8.95	–	−8.45	–
Glyma.07G004300.4	Ca^2+^-transporting ATPase	9.30	–	–	–
Glyma.15G167500.4	Ca^2+^-transporting ATPase	8.15	–	–	–
Glyma.11G048300.2	Ca^2+^-transporting ATPase	−7.52	–	–	–
Glyma.02G186100.2	Ca2 + −transporting ATPase	–	9.37	8.96	–
Glyma.19G136400.2	Ca2 + −transporting ATPase	–	−7.98	–	7.88
Glyma.05G108200.1	Extracellular signal-regulated kinase 1/2	9.60	5.28	–	–
Glyma.08G115300.1	Extracellular signal-regulated kinase 1/2	3.32	–	–	–
*Biotic*					
Glyma.15G206800.1	Chitinase	8.69	9.09	–	–
Glyma.12G156600.1	Chitinase	5.09	10.32	–	–
Glyma.19G245400.1	Chitinase	4.39	4.20	–	–
Glyma.17G076100.1	Chitinase	4.21	–	–	–
Glyma.02G042500.1	Chitinase	3.34	–	–	–
Glyma.11G124500.1	Chitinase	5.05	–	–	–
Glyma.16G173000.1	Chitinase	3.53	–	−3.07	–
Glyma.13G346700.1	Chitinase	4.58	–	–	–
Glyma.15G143600.1	Chitinase	−3.19	–	–	–
Glyma.15G062500.1	Pathogenesis-related protein 1	3.57	–	–	–
Glyma.15G062400.1	Pathogenesis-related protein 1	3.75	–	–	–
Glyma.13G094200.1	Pathogenesis-related protein 1	4.19	–	–	
*biotic. Proteinase inhibitors*					
Glyma.12G234800.1		6.24	3.97	–	3.12
Glyma.08G341700.1		4.10	4.72	–	3.18
Glyma.08G341300.1		9.65	6.16	–	3.49
Glyma.09G163900.1		6.78	5.46	–	–
Glyma.08G235400.1		9.47	7.27	–	–
Glyma.08G341400.1		9.18	6.00	–	–
Glyma.16G212400.1		9.90	7.93	–	–
Glyma.09G163700.1		7.37	–	–	–
Glyma.02G156800.1	Lectin, mannose-binding 2	7.06	4.28	–	–
*Isoprenoid*					
Glyma.08G277000.1	1-Deoxy-D-xylulose-5-phosphate synthase	5.15	–	–	–
Glyma.01G134600.4	Homogenitisate phytyltransferase	5.30	2.40	–	–
Glyma.10G070200.1	Homogenitisate phytyltransferase	6.62	–	–	–
Glyma.02G188200.3	Prolycopene isomerase	7.49	–	−8.17	–
Glyma.12G197400.1	Isoprene synthase	9.73	9.14	–	–
Glyma.06G302200.1	Isoprene synthase	3.07	6.08	–	–
Glyma.13G326400.2	(E)-4-hydroxy-3-methylbut-2-enyl-diphosphate synthase	–	8.05	–	–
Glyma.14G004600.2	Acetyl-CoA C-acetyltransferase	–	9.16	–	−8.16
Glyma.15G121400.2	Farnesyl diphosphate synthase	–	7.72	–	–
*Phenylpropanoid*					
Glyma.03G181700.1	Phenylalanine ammonia-lyase	4.14	–	–	–
Glyma.02G309300.1	Phenylalanine ammonia-lyase	4.83	–	–	–
Glyma.19G182300.1	Phenylalanine ammonia-lyase	3.10	–	–	–
Glyma.01G004200.4	Caffeoyl-CoA O-methyltransferase	9.08	–	−8.20	–
Glyma.05G147000.1	Caffeoyl-CoA O-methyltransferase	8.73	–	–	–
Glyma.01G187700.1	Caffeoyl-CoA O-methyltransferase	4.32	–	–	–
Glyma.09G281800.1	Caffeic acid 3-O-methyltransferase	5.28	6.62	–	−3.63
Glyma.09G281900.1	Caffeic acid 3-O-methyltransferase	8.60	4.37	–	–
Glyma.07G048900.1	Caffeic acid 3-O-methyltransferase	3.50	–	–	–
Glyma.01G021000.1	Cinnamyl-alcohol dehydrogenase	9.87	8.21	–	–
Glyma.13G255300.1	Cinnamyl-alcohol dehydrogenase	–	7.74	–	−7.74
Glyma.14G221200.1	Cinnamyl-alcohol dehydrogenase	–	–	−2.96	–
Glyma.04G039900.1	Shikimate O-hydroxycinnamoyl transferase	9.00	–	–	–
Glyma.02G283500.1	Shikimate O-hydroxycinnamoyl transferase	–	–	5.69	5.44
Glyma.08G220200.3	Shikimate O-hydroxycinnamoyl transferase	–	–	–	−4.63
Glyma.13G302500.1	Shikimate O-hydroxycinnamoyl transferase	–	–	−4.56	–
Glyma.04G040400.1	Shikimate O-hydroxycinnamoyl transferase	–	–	−4.76	–
Glyma.18G103500.1	Shikimate O-hydroxycinnamoyl transferase	–	–	5.88	–
Glyma.18G267800.1	Trans-resveratrol di-O-methyltransferase	4.35	–	–	7.24
Glyma.10G176500.1	Trans-resveratrol di-O-methyltransferase	7.73	–	–	–
*Flavonoid*					
Glyma.10G292200.1	Chalcone isomerase	6.19	3.67	–	–
Glyma.20G241700.1	Chalcone isomerase	3.07	–	–	–
Glyma.20G241500.2	Chalcone isomerase	7.86	–	–	–
Glyma.06G143000.1	Chalcone isomerase	3.02	–	–	–
Glyma.08G110300.1	Chalcone synthase	8.63	–	–	–
Glyma.08G109500.1	Chalcone synthase	5.15	–	–	–
Glyma.09G075200.1	Chalcone synthase	4.29	–	–	9.44
Glyma.01G228700.1	Chalcone synthase	4.90	–	–	–
Glyma.11G011500.1	Chalcone synthase	3.38	–	–	–
Glyma.01G091400.1	Chalcone synthase	5.45	–	–	–
Glyma.08G110900.1	Chalcone synthase	11.16	–	–	–
Glyma.08G110500.1	Chalcone synthase	5.13	–	–	–
Glyma.09G269600.1	Bifunctional dihydroflavonol 4-reductase/flavanone 4-reductase	5.99	3.67	–	–
Glyma.18G220600.1	Bifunctional dihydroflavonol 4-reductase/flavanone 4-reductase	3.97	–	–	–
Glyma.09G269500.1	Bifunctional dihydroflavonol 4-reductase/flavanone 4-reductase	4.08	–	–	–
Glyma.11G070500.1	Leucoanthocyanidin reductase	5.91	4.47	–	–
Glyma.01G211800.1	Leucoanthocyanidin reductase	8.88	–	–	–
Glyma.01G172700.1	Leucoanthocyanidin reductase	4.78	–	–	–
Glyma.11G070200.2	Leucoanthocyanidin reductase	4.57	–	–	–
Glyma.16G103900.1	Leucoanthocyanidin reductase	3.50	–	–	–
Glyma.01G172900.3	Leucoanthocyanidin reductase	4.62	–	–	–
Glyma.01G172600.1	Leucoanthocyanidin reductase	6.37	–	–	–
Glyma.04G131100.1	Leucoanthocyanidin reductase	–	9.27	–	–
*Cytochrome P450*					
Glyma.11G062500.1	Cytochrome P450, family 71, subfamily D, polypeptide 9 (flavonoid 6-hydroxylase)	5.95	–	–	–
Glyma.11G062600.1	Cytochrome P450, family 71, subfamily D, polypeptide 9 (flavonoid 6-hydroxylase)	6.49	–	–	–
Glyma.18G080400.1	Cytochrome P450, family 71, subfamily D, polypeptide 9 (flavonoid 6-hydroxylase)	7.66	–	–	–
Glyma.20G008200.4	Cytochrome P450, family 71, subfamily D, polypeptide 9 (flavonoid 6-hydroxylase)	–	–	7.52	–
Glyma.07G083000.1	Cytochrome P450, family 76, subfamily M, polypeptide 7 (ent-cassa-12,15-diene 11-hydroxylase)	3.74	–	–	–
Glyma.11G197300.1	Cytochrome P450, family 79, subfamily A, polypeptide 2 (phenylalanine N-monooxygenase)	6.76	7.50	–	–
Glyma.03G030400.1	cytochrome P450, family 83, subfamily B, polypeptide 1	–	–	−5.19	–
Glyma.03G129200.1	cytochrome P450, family 86, subfamily A, polypeptide 1 (fatty acid omega-hydroxylase)	–	–	–	5.55
Glyma.08G125100.1	Cytochrome P450, family 90, subfamily B, polypeptide 1 (steroid 22-alpha-hydroxylase)	−7.49	–	–	–
Glyma.03G143700.1	Cytochrome P450, family 93, subfamily A, polypeptide 1 (3,9-dihydroxypterocarpan 6a–monooxygenase)	5.07	–	–	–
Glyma.19G144700.1	Cytochrome P450, family 93, subfamily A, polypeptide 1 (3,9-dihydroxypterocarpan 6a–monooxygenase)	4.42	–	–	–
Glyma.13G173500.1	Cytochrome P450, family 93, subfamily C (2-hydroxyisoflavanone synthase)	3.76	–	–	–
Glyma.17G227500.1	Cytochrome P450, family 97, subfamily A (beta-ring hydroxylase)	8.58	–	–	–
Glyma.03G226800.3	Cytochrome P450, family 734, subfamily A, polypeptide 1 (PHYB activation tagged suppressor 1)	6.24	9.44	–	–
*Simple phenol*					
Glyma.U027300.1	L-ascorbate oxidase	8.27	7.49	–	–
Glyma.01G108200.1	L-ascorbate oxidase	5.84	–	–	–
Glyma.07G142600.1	L-ascorbate oxidase	4.88	–	–	–
Glyma.18G193400.1	L-ascorbate oxidase	5.53	–	–	
*Transcription factors*					
Glyma.16G054400.1	WRKY transcription factor 33/WRKY	5.32	–	–	
Glyma.15G186300.1	WRKY transcription factor 33/WRKY	8.95	–	–	–
Glyma.02G141000.5	WRKY transcription factor 22/WRKY	–	8.16		–
Glyma.12G221500.1	NAC	10.01	5.16	–	–
Glyma.12G149100.1	NAC	6.66	6.43	–	–
Glyma.08G173400.1	NAC	3.57	3.63	–	–
Glyma.16G151500.1	NAC	5.80	–	–	–
Glyma.09G032100.1	Myb proto-oncogene protein, plant/MYB	6.50	–	–	–
Glyma.11G142900.1	Myb proto-oncogene protein, plant/MYB	5.94	–	–	–
Glyma.16G006500.9	MYB	7.83	–	–	−7.57
Glyma.13G144600.3	Myb proto-oncogene protein, plant/MYB	–	7.91	–	–
Glyma.10G180800.1	myb proto-oncogene protein, plant/MYB	–	–	–	−4.06
Glyma.19G104200.1	EREBP-like factor/AP2-EREBP	8.49	–	–	–
Glyma.10G036600.1	Ethylene receptor/AP2-EREBP	4.31	5.95	–	–
Glyma.10G036700.1	Ethylene-responsive transcription factor 1/AP2-EREBP	4.63	7.78	–	–
Glyma.10G036600.1	EREBP-like factor/AP2-EREBP	4.31	5.95	–	–
Glyma.10G223200.1	EREBP-like factor/AP2-EREBP	8.24	–	–	–
Glyma.07G212400.1	EREBP-like factor/AP2-EREBP	−4.88	–	5.52	–
Glyma.16G164800.1	EREBP-like factor/AP2-EREBP	7.76	–	–	–
Glyma.10G061400.1	EREBP-like factor/AP2-EREBP	−8.71	–	–	–
Glyma.19G248900.2	Ethylene-responsive transcription factor 1/AP2-EREBP	–	8.36	–	–
Glyma.19G248900.1	Ethylene-responsive transcription factor 1/AP2-EREBP	–	4.98	–	–
Glyma.13G329700.2	AP2-like factor, euAP2 lineag/AP2-EREBP	–	–	−6.95	–
Glyma.18G159900.2	AP2-EREBP	–	–	–	−7.67

*HRK* represented the highly resistant line Gantai-2-2; *HSK* represented the highly susceptible line Wan82–178; and the numbers 0 and 48 represented the processing times

Under biotic and abiotic stresses, a great number of ROS in plants will be produced, and the cell structure is then destroyed. Plants often remove ROS in vivo through the production of a reactive oxygen scavenging agent, in order to alleviate damage to the plants caused by oxidative stress [13]. ROS removal system mainly includes enzymes, such as POD and PPO, and low molecular weight antioxidants, such as ferredoxin and thioredoxin (TRX) [14, 15]. After bean pyralid larvae feeding for 48 h, many genes related to the ROS removal system were identified that were significantly up-regulated (Tables 2 and 3). For example, there were 23 POD, 5 PPO, 9 glutathione S-transferase (GST) and 3 TRX1 identified in Gantai-2-2. Additionally, there were 20 POD, 4 PPO and 2 GST identified in Wan82–178. When comparing Gantai-2-2 with Wan82–178 at 0 h feeding, 1 GST was down-regulated. When comparing Gantai-2-2 with Wan82–178 at 48 h feeding, 1 TRX1 was up-regulated.

JA, ethylene (ET), and other plant hormone signaling pathways can be activated after pest feeding, which in turn causes a rise in the defense gene expression levels, an accumulation of defensive compounds, and an increase in the release of volatiles, finally, the plants showed resistance to the pests [16–18]. Our results showed that the genes related to plant hormone had changed after bean pyralid feeding, including JA, ET and auxin (Tables 2 and 3). Eight DEGs related to JA biosynthesis were all up-regulated in Gantai-2-2, including 1 lipoxygenase (LOX), 3 linoleate 9S–LOX, 1 alpha-dioxygenase (α-DOX), 1 hydroperoxide dehydratase and 2 12-oxophytodienoic acid reductase (OPDA). Additionally, 6 DEGs related to JA biosynthesis were all up-regulated in Wan 82–178, including 1 LOX, 2 linoleate 9S–LOX, 1 α-DOX, 1 hydroperoxide alpha dehydratase and 1 OPDA. When comparing Gantai-2-2 with Wan82–178 at 0 h feeding, 1 linoleate 9S–LOX and 1 OPDA were down-regulated. When comparing Gantai-2-2 with Wan82–178 at 48 h feeding, 1 LOX and 1 OPDA were down-regulated. There were 24 DEGs related to ET biosynthesis and signal transduction identified in Gantai-2-2, of which 22 DEGs were significantly up-regulated, including 7 aminocyclopropane carboxylate oxidase (ACC oxidase), 2 ethylene receptors, 3 ethylene responsive factor 1 (ERF1), 5 EREBP-like factor, and so on. Additionally, 14 DEGs related to ET biosynthesis and signal transduction identified in Wan 82–178 were all significantly up-regulated, including 5 ACC oxidase, 2 ethylene receptors, 2 EREBP-like factor, 3 ERF1, and so on. One ACC oxidase was found to be higher in Gantai-2-2 than in Wan82–178 at 0 h. In addition, 1 EREBP-like factor was found to be higher in Gantai-2-2 than in Wan82–178 at 48 h. There were 12 significantly up-regulated DEGs associated with auxin synthesis and signal transduction pathways in Gantai-2-2, including 4 IAA-amino acid hydrolase, 1 auxin responsive GH3 gene family, 3 SAUR family proteins, and so on. Six DEGs associated with auxin synthesis and signal transduction pathways identified in Wan82–178 were significantly up-regulated, including 1 IAA-amino acid hydrolase, 3 SAUR family protein, and so on. In addition, 1 SAUR family protein was found to be higher in Gantai-2-2 than in Wan82–178 at 48 h.

The results showed that many genes related to protein kinase could be induced by bean pyralid (Tables 2 and 3). After bean pyralid feeding for 48 h, 17 protein kinases were identified in Gantai-2-2, that were significantly up-regulated, including 2 protein kinase, 1 protein kinase A, 5 LRR receptor-like serine/threonine-protein kinase FLS2, 3 serine/threonine-protein kinase PBS1 (STK), 2 serine/threonine-protein kinase SRK2, 1 serine/threonine-protein kinase WNK1 and 3 serine/threonine-protein kinase CTR1. Additionally, 7 protein kinases were identified in Wan82–178, of which 6 DEGs were up-regulated, including 1 protein kinase A, 2 LRR receptor-like serine/threonine-protein kinase FLS2, 2 serine/threonine-protein kinase PBS1 (STK) and 1 serine/threonine-protein kinase WNK1. The results showed that many genes related to Ca^2+^ could be induced by bean pyralid (Table 3). After bean pyralid feeding for 48 h, 7 DEGs associated with Ca^2+^ signaling were identified in Gantai-2-2, of which 6 DEGs were up-regulated, including 1 calmodulin, 2 calcium-binding protein CML and 3 Ca^2+^-transporting ATPase. Additionally, 8 DEGs associated with Ca^2+^ signaling were identified in Wan82–178, of which 3 DEGs were up-regulated, including 1 calcium/calmodulin-dependent protein kinase, 1 calcium-binding protein CML and 1 Ca^2+^-transporting ATPase.

Some genes induced by abiotic stress may have been induced by insects as well, for example, the genes associated with harm and drought stresses are often induced by chewing insects [19–21]. Meanwhile, Our results showed that many genes related to biotic and abiotic stresses were induced by bean pyralid too. After bean pyralid feeding for 48 h, many genes related to biotic stress could be induced by bean pyralid (Tables 2 and 3). For example, 43 DEGs related to biotic stress were identified in Gantai-2-2, of which 39 DEGs were up-regulated, including 12 PR-proteins, 8 proteinase inhibitors, 9 chitinase, 1 lectin mannose-binding 2, and so on. Additionally, 22 DEGs were associated with biotic stress identified in Wan82–178, of which 20 DEGs were up-regulated, including 7 PR-proteins, 7 proteinase inhibitors, 3 chitinase, 1 lectin mannose-binding 2, and so on. In addition, 3 proteinase inhibitors were found to be higher in Gantai-2-2 than in Wan82–178 at 48 h. After bean pyralid feeding for 48 h, 23 DEGs associated with abiotic stress, such as pathogen infection, heat stress, cold stress and drought stress, were identified in Gantai-2-2, of which 17 DEGs were up-regulated. Additionally, 16 DEGs associated with abiotic stress were identified in Wan82–178, of which 9 DEGs were up-regulated (Tables 2 and 3). When comparing Gantai-2-2 with Wan82–178 at 0 h feeding, 3 DEGs associated with abiotic stress were up-regulated and 9 DEGs were down-regulated. When comparing Gantai-2-2 with Wan82–178 at 48 h feeding, 3 DEGs associated with abiotic stress were up-regulated and 3 DEGs were down-regulated.

It has widely been reported that secondary metabolism pathways, such as isoprenoid, phenylpropanoid, flavonoid, cytochrome P450 and simple phenol metabolism, were involved in plant resistance to insect feeding, or possibly functioned as direct defense compounds, antioxidants, signaling molecules or insect toxins [22–24]. Our results showed that many genes related to secondary metabolism pathways could be induced by bean pyralid (Tables 2 and 3). After bean pyralid feeding for 48 h, 6 DEGs related to isoprenoid biosynthesis pathway were identified in Gantai-2-2 that were all up-regulated, including 1 1-deoxy-D-xylulose-5-phosphate synthase (DXS), 2 homogentisate phytyltransferas (HPT), 1 prolycopene isomerase and 2 isoprene synthase. Additionally, 6 DEGs were identified in Wan82–178 that were all up-regulated, including 1 HPT, 2 isoprene synthase, 1 (E)-4-hydroxy-3-methylbut-2-enyl-diphosphate synthase, 1 acetyl-CoA C-acetyltransferase and 1 farnesyl diphosphate synthase. When comparing Gantai-2-2 with Wan82–178 at 0 h feeding, 1 prolycopene isomerase was down-regulated. When comparing Gantai-2-2 with Wan82–178 at 48 h feeding, 1 acetyl-CoA C-acetyltransferase was down-regulated. There were 13 DEGs associated with phenylpropanoid biosynthesis pathway were identified in Gantai-2-2 that were all up-regulated, including 3 phenylalanine ammonia-lyase (PAL), 3 caffeoyl-CoA 3-O-methyl transferase (CCOAOMT), 3 caffeic acid 3-O-methyltransferase (COMT), 1 cinnamyl-alcohol dehydrogenase (CAD), 2 trans-resveratrol di-O-methyltransferase and 1 shikimate O-hydroxycinnamoyl transferase. Additionally, 4 DEGs were identified in Wan82–178 were all up-regulated, including 2 COMT and 2 CAD. When comparing Gantai-2-2 with Wan82–178 at 0 h feeding, 1 CCOAOMT, 1 CAD and 2 shikimate O-hydroxycinnamoyl transferase were down-regulated, and 2 shikimate O-hydroxycinnamoyl transferase were up-regulated. When comparing Gantai-2-2 with Wan82–178 at 48 h feeding, 1 COMT, 1 CAD and 1 shikimate O-hydroxycinnamoyl transferase were down-regulated, and 2 shikimate O-hydroxycinnamoyl transferase was up-regulated. There were 22 DEGs associated with flavonoid biosynthesis pathway identified in Gantai-2-2 that were all up-regulated, including 4 chalcone isomerase (CHI), 8 chalcone synthase (CHS), 3 dihydroflavonol4-reductase (DFR) and 7 leucoanthocyanidin reductase (LAR). Additionally, 4 DEGs were all up-regulated identified in Wan82–178, including 1 CHI, 1 DFR and 2 LAR. When comparing Gantai-2-2 with Wan82–178 at 48 h feeding, 1 CHS was up-regulated. After bean pyralid feeding for 48 h, 26 DEGs related to cytochrome P450 (CPY) were identified in Gantai-2-2, of which 25 DEGs were up-regulated. Additionally, 8 DEGs were identified in Wan82–178, of which 7 DEGs were up-regulated. When comparing Gantai-2-2 with Wan82–178 at 0 h feeding, 1 CPY was up-regulated and 1 CPY was down-regulated. When comparing Gantai-2-2 with Wan82–178 at 48 h feeding, 1 CPY was up-regulated. After bean pyralid feeding for 48 h, 4 and 1 L-ascorbate oxidase (L-AO) were identified in Gantai-2-2 and Wan82–178, respectively, which were all up-regulated.

### Bean pyralid-induced the transcription factor genes

Transcription factors, known as trans-acting factors, are a type of DNA binding protein that regulates the transcription of the target genes by combining with cis-acting elements in the gene promoter [25]. Research demonstrated that plant transcription factors, including NAC, MYB, WRKY, were involved in the plant defense responses [26]. Our results showed that 25 transcription factors were identified in Gantai-2-2, including 4 NAC, 7 AP2-EREBP, 3 MYB, 2 WRKY, 1 bHLH, 2 C3H, and so on, of which 21 were up-regulated and 4 were down-regulated, after bean pyralid larvae feeding for 48 h (Table 4). Fifteen transcription factors were identified in Wan82–178, including 3 NAC, 4 AP2-EREBP, 1 MYB, 1 WRKY, 2 bHLH, 3 C3H, and so on, which were all up-regulated, after bean pyralid larvae feeding for 48 h. When comparing Gantai-2-2 with Wan82–178 at 0 h feeding, 7 transcription factors were identified, of which 1 AP2-EREBP, 1 C2C2-YABBY and 1 FAR1 were up-regulated, and 1 AP2-EREBP, 2 MYB and 1 LIM were down-regulated. When comparing Gantai-2-2 with Wan82–178 at 48 h feeding, 5 transcription factors were identified, of which 1 FAR1 was up-regulated, and 1 AP2-EREBP, 2 MYB and 1 C3H were down-regulated (Table 4).

**Table 4:** Transcription factors (TF) related to the insect resistance identified in different alignment schemes

TF family	HRK48/HRK0		HSK48/HSK0		HRK0/HSK0		HRK48/HSK48	
	up	down	up	down	up	down	up	down
NAC	4	0	3	0	0	0	0	0
PLATZ	1	0	0	0	0	0	0	0
AP2-EREBP	5	2	4	0	1	1	0	1
MYB	3	0	1	0	0	2	0	2
bHLH	1	0	2	0	0	0	0	0
WRKY	2	0	1	0	0	0	0	0
C3H	2	0	3	0	0	0	0	1
C2H2	1	0	0	0	0	0	0	0
LIM	1	0	0	0	0	0	0	0
TUB	0	1	0	0	0	1	0	0
GRF	1	0	0	0	0	0	0	0
ARF	0	1	0	0	0	0	0	0
Tify	0	0	1	0	0	0	0	0
C2C2-YABBY	0	0	0	0	1	0	0	0
FAR1	0	0	0	0	1	0	1	0
Sum	21	4	15	0	3	4	1	4

*HRK* represented the highly resistant line Gantai-2-2; *HSK* represented the highly susceptible line Wan82–178; numbers 0 and 48 represented the processing time

### Analysis of DEGs by qRT-PCR

**Fig. 8:**
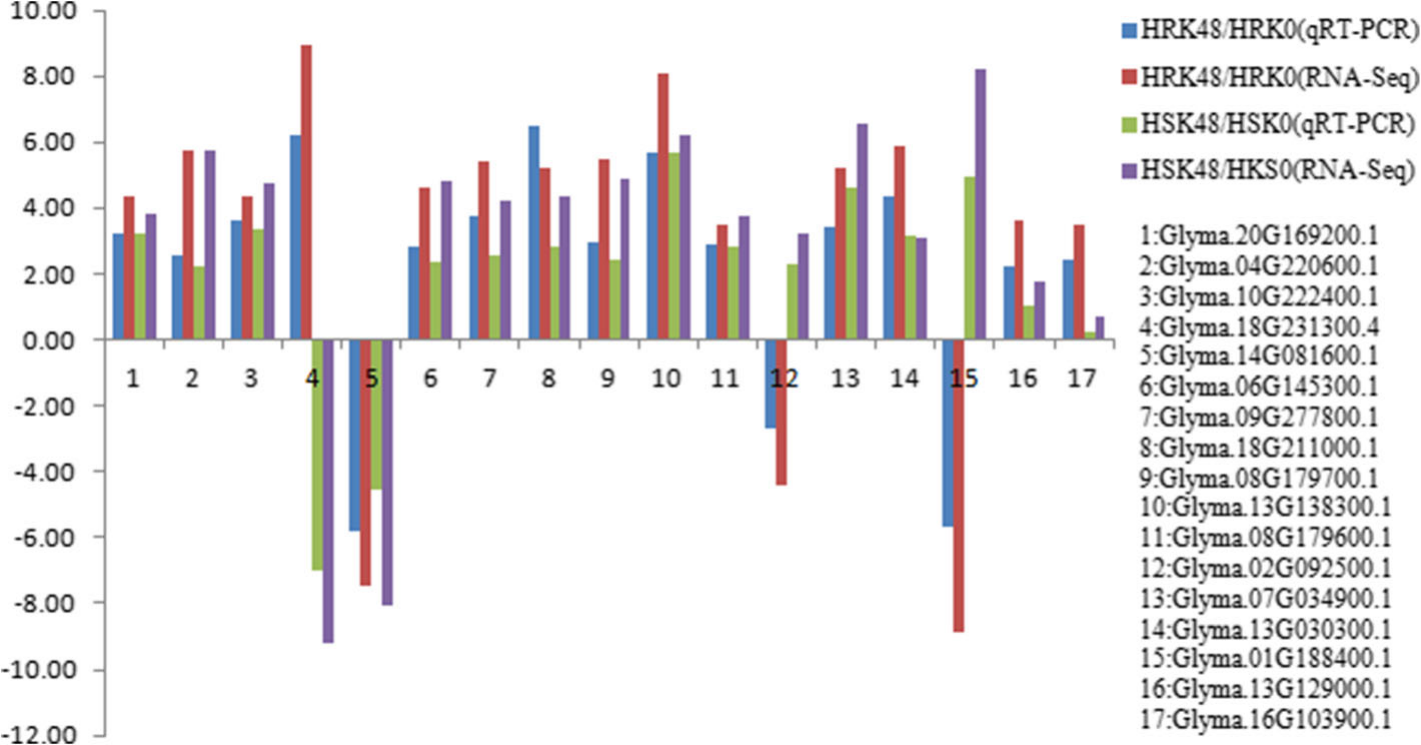
DEGs confirmed by qRT-PCR using the same sample as that in RNA-Seq. X-axis represented gene name, the blue column represented qRT-PCR results in HRK48/HRK0, the red column represented RNA-Seq results in HRK48/HRK0, the green column represented qRT-PCR results in HSK48/HSK0, and the purple column represented RNA-Seq results in HSK48/HSK0; Y-axis represented the relative level of gene expression

The quantitative real time-PCR (qRT-PCR) technology was used to verify the credibility of the RNA-Seq. Therefore, we used the qRT-PCR technology to verify 17 DEGs identified by RNA-Seq. It was found that the qRT-PCR expression patterns of 17 DEGs were all consistent with the RNA-Seq results (Fig. 8), which indicated that the RNA-Seq results were reliable in the present study.

## Discussion

### Influence of the genes related to ROS removal on bean pyralid response

As an electron acceptor of H_2_O_2_, POD oxidizes various materials in the process of secondary metabolites and can produce phenoxy and oxygen free radicals by interacting with some phenolic compounds, which may also directly interfere with the feeding of insects or reduce the nutrition level of leaves, thereby lowering the edibility of the plants [27]. Meanwhile, POD has been found to have significant insecticidal effects on Lepidoptera and Coleoptera [28]. Previous studies have shown that POD could be induced by insects in wheat [29], sorghum [30], cucumber [31] and rice [32, 33]. PPO is widely distributed in plants and can potentially catalyze the oxidation of polyphenols into a keto metal enzyme, it can directly oxidize acid into quinone using O_2_ as an oxidation substrate, then produce amino acids and proteins that are difficult to digest and toxic to herbivores. Meanwhile, as an anti-nutritional protein, PPO can produce protein affinity, and therefore reduce the edibility of plants, which plays an important role in the defense of insect feeding [34, 35]. Previous research studies have shown that the activity of PPO increased after tomatoes were eaten by *Spodoptera exigua* or were treated with oral secretions of *Spodoptera exigua* [36, 37]. GST is encoded by a large and diverse family of genes, that plays important roles in the responses of plants to oxidative damage induced by various environmental conditions, especially in the resistance to resist the toxic effects of ROS on cells [38–40]. In oxygen stress reactions, TRX would pass restoring forces to the reductase, which could potentially remove the lipid peroxide or repair the oxidized protein, resulting in an alleviation of oxygen stress [41]. TRXh5 and TRXh8 in *Arabidopsis thaliana* were strongly induced under biotic or abiotic stress [42, 43]. After bean pyralid larvae feeding for 48 h, we found that POD, PPO and GST were all significantly up-regulated in Gantai-2-2 and Wan82–178. These results suggested that these genes were involved in the defense reactions of soybean to bean pyralid. TRX1 was up-regulated in Gantai-2-2, which suggested that TRX1 involved in defense responses to bean pyralid in the highly resistant material only.

### Influence of plant hormones on bean pyralid response

JA signaling pathway is implicated in insect-induced responses in plant; it was involved in the signal transduction of insect defense, regulated the expression of plant downstream defense genes, and significantly induced responses of defense systems in plant, thereby effectively reducing pests [44]. After bean pyralid feeding, 3 key genes involved in JA biosynthetic pathway were identified in Gantai-2-2 and Wan82–178, namely, LOX, *α*-DOX and OPDA, which were significantly up-regulated. LOX is the key enzyme in the synthesis of the JA [45], and plays an important signal factor in plant induction defense pathways [46]. Adversity stresses, such as insects and diseases, could induce single or multiple LOX genes in plants [47, 48]. Additionally, *α*-DOX could catalyze the oxidation of fatty acids and produce 2-hydrogen peroxide fatty acids, and it is also an enzyme related to plant stress resistance in JA biosynthetic pathway [32]. For example, *α*-DOX can be induced by insects in rice [32, 49]. OPDA is the intermediate product of JA biosynthesis pathway with biological activities that can regulate plant defense genes. For example, OPDA has been found to induce defense genes in *Arabidopsis thaliana* which resist attacks from *Bradysia odoriphaga* [50]. As a plant endogenous hormone, ET regulates the defense responses of plants to pests and diseases [51, 52]. Our results found that 3 types of genes related to ET biosynthesis and signal transduction pathway. For example, most of ACC oxidase, ethylene receptor and ERF1 genes were up-regulated after bean pyralid feeding. ACC oxidase is one of the key enzymes in ethylene biosynthesis pathway [52]. Ethylene receptor is an upstream component of ethylene signal transduction pathway, that plays an important role in plant growth and responses to adversity stresses [53]. ERF is a specific plant transcription factor that plays an important role in plant cell growth, hormone regulation, disease resistance and abiotic stresses [54–56]. Auxin can directly act on cell membranes or intracellular components, and affects some cellular responses. In addition, it can also indirectly regulate the expression of genes, and has a direct impact on plant stress [57, 58]. After bean pyralid feeding, some genes related to auxin synthesis and signal transduction pathways were identified, such as IAA-amino acid hydrolase, auxin responsive GH3 (Gretchen Hagen3) gene family and SAUR (small auxin up RNA) family protein. Early/primary auxin response genes were composed of three major gene families: Aux/IAA (auxin/indoleacetic acid), GH3 and SAUR transcription factor family [59]. GH3 gene family is a typical auxin-responsive gene family and can promote amino acids to be combined with IAA, JA and SA, then changed the concentration of biologically active forms within the cells. This aided in regulating plant growth, development and defense responses [60]. SAUR gene family is known to be the specific and largest family among the auxin response factors in plants and is related to environmental stimuli [61, 62]. These results indicated that JA, ET and auxin may be involved in elevating the basal resistance of soybean to herbivory.

### Influence of the intracellular signal transduction pathway on bean pyralid response

Protein kinase is involved in the signal transduction pathway of plants and plays an important role in the defense responses of plants [63]. Protein kinases could be induced by bean pyralid, including protein kinase, protein kinase A, LRR receptor-like serine/threonine-protein kinase FLS2, serine/threonine-protein kinase PBS1 (STK), serine/threonine-protein kinase SRK, serine/threonine-protein kinase WNK1, and serine/threonine-protein kinase CTR1. LRR receptor-like kinase is the largest family of receptor protein kinase in plants; it plays an important role in the regulation of plant growth, development, biotic and abiotic stresses [64]. Serine/threonine-protein kinase (STK) is an important signal molecule; when plant suffers stimulation, such as pest feeding, salinity, drought stress, trauma, cytokines or hormones, STK is rapidly activated in the serine and threonine residue phosphorylation sites and is further activated in the downstream signal molecules through phosphorylation cascades, which activate specific signaling pathways. It is eventually transmitted by an outside signal to the nucleus, and activates or inhibits specific genes [64–66]. These results indicated that protein kinases played an important role in the defense against bean pyralid.

When the plants were stimulated by external environmental and received the signal, the receptor activated the calcium channels on the membrane through a series of phosphorylation reactions to cause calcium ions to be released from the calcium base into the cytoplasm. This response led to an increase in the concentration of Ca^2+^ in the cytoplasm, which then activated the plant defense response [67, 68]. Previous studies have shown that Ca^2+^ could be induced by S*podoptera littoralis* in *Phaseolus lunatus* [69] and *Ginkgo biloba* [70]. After bean pyralid feeding, most of the DEGs related to Ca^2+^ signaling identified in Gantai-2-2 were up-regulated, but in Wan82–178 were down-regulated. It was suggested that Ca^2+^ signaling was involved in the defense responses to bean pyralid in the highly resistant material. After soybean was stimulated by bean pyralid, the concentrations of Ca^2+^ in the cytoplasm changed; therefore, calcium was sent to transfer the stimulation signal. However, Ca^2+^ signaling may have played a negative role in the regulation of defense responses in the highly susceptible material.

### Influence of the genes related to biotic stress on bean pyralid response

When plants suffer from pest stress, a variety of defense proteins which inhibit the insect from producing digestive enzymes are produced to resist insect pests, including PR, proteinase inhibitors, chitinase and lectin [71, 72] and thereby destroy the insect’s normal digestive absorption functions, then disrupt their nutrient uptake and utilization, which could ultimately lead to malnutrition and inhibited growth of the insects [73].

PR proteins are generated and accumulated after pathogen invasions or abiotic stress and are inducible components in the self defense mechanisms of plants [74–76]. Uknes et al. found that the contents of PR-1, PR-2 and PR-5 were increased, when the *Arabidopsis thaliana* were treated with TCV and INA, and its disease resistance was increased [77]. After bean pyralid feeding, most of the DEGs related to PR were induced. Our results speculated that the PR proteins might be involved in the defense responses of soybean to bean pyralid.

Proteinase inhibitors can inhibit the activity of chymotrypsin and trypsin in the digestive tract of the Lepidoptera and Diptera, to inactivate the related digestive enzyme in intestines, and thereby interfering with the insects’ normal feeding and digestion. Therefore, the insects cannot attain enough nutrition, which in turn affects their growth and development, then leads to insect death [78, 79]. In other cases, it caused excessive production of proteases in insects, which resulted in a deficit of essential amino acids, then caused insect death [80]. Our results showed that the proteinase inhibitors could be induced by bean pyralid, so we assumed that the proteinase inhibitors were involved in the defense responses.

Chitinase could destroy the peritrophic membranes of insects [81]. When insect eats plants with sustained chitinase expression, its digestive tract becomes damaged and its epidermis cannot form normally [82]. Chitinase could be induced by aphid and spider mites in sorghum [83] and tomato [84], respectively. Our results showed that chitinase could be induced by bean pyralid as well. It was assumed that after the chitinase was absorbed into the insects’ bodies, chitin was hydrolyzed, which inhibited the growth and development of insects and achieved the purpose of defense for the plants [85].

Lectin has at least one non-catalytic domain-specific reversible binding to monosaccharides or oligosaccharides, and after being absorbed into the gut of an insect, lectin induces local or systematic toxic effects, which bond to glycoproteins on the peritrophic membrane and damage the structure of the peritrophic membrane. This response causes insect growth arrest, antifeedant and even death [86, 87]. Previous results have shown that lectin displays insecticidal activities against a variety of insects. For example, after *Heliothis virescens* [88], *Myzus persicae* [89] and *Lacanobia oleracea* [90] fed on lectin-transgenic plants, their survival rates and fecundity were greatly reduced. Our results showed that the lectin, mannose-binding 2 could be induced by bean pyralid. It was assumed that the lectin that was released from damaged insect cells of the soybean combined with the chitin of the peritrophic membranes, sugar compounds of the digestive tract epithelial cells, and the glycosylation digestive enzymes, after bean pyralid feeding. This affected the normal absorption of nutritional materials. It also induced disease in the digestive tracts and promoted gastrointestinal bacterial reproduction, thereby causing insect growth inhibition or death, which achieved the defensive purpose of killing the pests [91].

### Influence of the genes related to the secondary metabolism on bean pyralid response

Isoprenoid biosynthesis pathway is an important secondary metabolism route that widely exists in plants. It has the ability to synthetize isoprenoid compounds, and plays an important role in plant resistance reactions, in addition to participating in the growth and development of plants [92]. The genes related to isoprenoid biosynthesis pathway could be induced by bean pyralid, which speculated that isoprenoid biosynthesis pathway was involved in the defense reactions of soybean to bean pyralid.

Phenylpropanoid and its derivatives play an important role in plant development and stress responses. Environmental stresses can promote plant carbon synthesis, which changes into benzene propane synthesis, causing the accumulations of large amounts of substances related to plant stress resistance, such as lignin, flavone, flavonol and alkaloids. These substances have been found to improve the resistance of plants to various biotic and abiotic stresses [93]. The genes related to phenylpropanoid biosynthesis pathway, including PAL, CCOAOMT, COMT and CAD, could be induced by bean pyralid. PAL is not only the enzyme that catalyzes the first step of phenylpropanoid biosynthesis pathway but also the key and rate limiting enzyme for the pathway. Various metabolite substances that are catalyzed by PAL, such as lignin, flavonoids and phytoalexin, were related to plant insect resistance [94]. PAL can be induced by many types of biotic and abiotic stresses, including pathogen infections, insect feeding, mechanical injuries and exogenous plant hormones [95–97]. CCOAOMT is a type of important methyltransferase in plant lignin biosynthesis [98, 99]. COMT catalyzes the reaction that converts caffeic acid into ferulic acid, which is an important step in the biosynthesis of lignin monomers [100]. CAD is the last step in lignin synthesis metabolic pathway and plays a key role in lignin biosynthesis [101, 102]. PAL, CCOAOMT, COMT and CAD were related to lignin, which suggested that soybean might have enhanced its cell mechanical strength through the synthesis of lignin, which reinforced the cell walls, strengthened the cell to blocked insect feeding, and defended the insect pests by nutrient limitation [103, 104].

Flavonoid plays an important role in insect-induced, disease, and other stress responses in plants [105]. The genes related to flavonoid biosynthesis pathway, including CHI, CHS, DFR and LAR, were found to be induced by bean pyralid. CHS and CHI are two key enzymes in flavonoid biosynthesis pathway; CHS catalyzes the condensation reaction of 4-coumaric acid-CoA and acyl-CoA, which causes chalcone to be formed, and flavonoid is then formed under the action of CHI, a necessary enzyme that syntheses flavanone, flavone, flavonol and anthocyanin substances [106, 107]. The amount of flavonoid metabolites was directly affected by the expression of CHS and CHI, and the loss of expression ability or loss of enzyme function [108]. DFR and LAR are key enzymes in flavonoid synthesis, and during the process of plant flavonoid biosynthesis, anthocyanins, proanthocyanidins and other common synthesis precursors are leucoanthocyanins. DFR was catalyzed by flavanonols to generate leucoanthocyanidin, LAR converted leucoanthocyanidin into 2,3-trans-flavan-3-ols [109]. Previous studies have shown that over-expressions of DFR could improve the content and antioxidant capacities of flavonoid [110]. Our results suggested that flavonoid biosynthesis pathway played an important role in the defense against bean pyralid and that flavonoid accumulation is an important characteristic of responses to abiotic stress in plants.

Cytochrome P450 (CYP) is a type of pheme containing oxidoreductases. It can catalyze some substances with a defensive function, such as sterol, isoflavone, alkaloid, terpenoid, and so on [111, 112]. CYP plays an important role in the defense of organisms against pests and abiotic stress [113]. CYP genes can be induced by aphid in soybean [114]. Our results showed that some genes related to CYP can also be induced by bean pyralid. Previous studies have shown that cyanogentic glycoside which was catalyzed and synthesized by the CYP79A and CYP71E1 genes in sorghum, was harmful to pests [115]. Therefore, it was speculated that soybean would use the CYP family to mitigate the threat of insect infestation.

AO is a copper-containing oxidase family that is wide spread in plants. It is a type of simple phenol and can catalyze the ascorbic acid (AA) of protoplasts in vitro and can oxygenate AA to generate monodehydroascorbate (MDHA), which regulates the overall oxidation state of the AA library of plant protoplasts in vitro [116, 117]. When activity of AO increased, the ratio of oxidation and reduction in extracellular AA was also increased. There was a significant redox gradient on both sides of the plasma membrane, which modulated plant defense responses against insect attack and other stressed [118–120]. Our results showed that some genes related to L-AO can also be induced by bean pyralid. Therefore, L-AO was involved in the response reactions of soybean to bean pyralid.

### Transcription factor on bean pyralid response

WRKY is one of the largest families of transcription factors in plants and plays an important role in the regulation of plant growth and development as well as biotic and abiotic stresses [121–123]. Previous studies have shown that WRKY could be induced by insect stress [124]. For example, one WRKY23 gene was induced by *Heterodera schachtii* in *Arabidopsis thaliana* [125] and 20 WRKY transcription factors were induced by cotton boll weevil in *Gossypium hirsutum* [126]. NAC is known to be a second major transcription factor in plants and is also a specific transcription factor. NAC can activate downstream genes when plants are exposed to biotic or abiotic stress, so it is involved in the stress responses of plants [127–129]. Some NAC genes were induced by insects in rape [130], sugarcane [131] and *Gossypium hirsutum* [126]. MYB is one of the central regulators of development, metabolism, and response to abiotic and biotic stresses [132]. One of the known mechanisms is that MYB transcription factor contribute to the accumulation of flavonoids to protect plants from radiation injury and enhance their resistance to cold and insect pests in plants [133]. MYB could be induced by small cabbage white caterpillars [134] and cotton boll weevil [126]. AP2/EREBP is a type of specific transcription factor which is bound with ethylene responsive elements. It is able to combine with the GCC box of the promoter region of some resistance related genes, and carry out the function of activating the expression of these genes [135]. AP2/EREBP can potentially regulate the molecular responses of plants to hormones, pathogens, low temperatures, drought and high salt, which could improve the tolerance of crops to stress [136–138]. For example, the over expression of *Tsi1* gene encoding EREBP/AP2 transcription factor increased the resistance to pathogens in tobacco, as well as the resistance ability to osmotic stress [138]. Our results showed that the transcription factor, such as WRKY, NAC, MYB and AP2/EREBP, could be induced by bean pyralid. It was confirmed that these transcription factors were involved in the defense reactions to bean pyralid.

## Conclusions

To explore the defense mechanisms of soybean resistance to bean pyralid, the comparative transcriptome sequencing was completed between the leaves infested with bean pyralid larvae and no worm of soybean (Gantai-2-2 and Wan82–178) on the Illumina HiSeq™ 2000 platform. The results showed that there were 1064 and 680 DEGs were identified in the Gantai-2-2 and Wan82–178 after bean pyralid larvae feeding for 48 h, respectively, compared to 0 h. When comparing Gantai-2-2 with Wan82–178, 605 DEGs were identified at 0 h feeding, and 468 DEGs were identified at 48 h feeding. The DEGs were divided into three categories, including the DEGs with non-bean pyralid-induced genotype, bean pyralid-induced DEGs that appeared in both materials and bean pyralid-induced genotype DEGs. According to GO and KEGG functional and metabolic pathway analysis combined with the previously reported literatures, we concluded that the response to bean pyralid feeding might be related to the disturbed functions and metabolism pathways of some key DEGs, such as DEGs involved in the ROS removal system, mainly POD, PPO, GST and TRX; phyto-hormone signaling pathways, mainly JA, ET and auxin pathway; intracellular signal transduction pathways, including plant protein kinases and Ca^2+^ signaling; secondary metabolism, such as isoprenoid biosynthesis pathway, phenylpropanoid biosynthesis pathway, flavonoid biosynthesis pathway, CYP and L-AO; transcription factors, such as WRKY, NAC, MYB and AP2/EREBP. Meanwhile, bean pyralid activated a large number of genes related to biotic and abiotic stresses. These results will help to elucidate the molecular mechanism of response to bean pyralid feeding in soybean, and provide a valuable resource of soybean defense genes that will benefit other studies in this field. Future research will focus on the cloning and transgenic function validation of possible candidate genes associated with the anti-bean pyralid soybean.

## Methods

### Plant materials

The tested materials Gantai-2-2 (highly resistant line) [3] and Wan82–178 (highly susceptible line) [3] were planted inside insect net rooms in experimental fields at the Guangxi Academy of Agricultural Sciences. The plants were sown in three rows, with 10 strains per row. During the entire growth period of soybeans, pesticides and fertilizers were not used. When the plants grew to a level of 10 compound leaves, the seventh compound leaves on the left side were collected before the infestation. There was a total of five plants for each sample. Then, each of the samples was simultaneously artificially infested with 5 four-year-old bean pyralids on the right side of the seventh leaves, counted downward. There were two biological repetitions. In each sample, five leaves were mixed, and they were frozen with liquid nitrogen and stored at −80 °C for further use.

### Total RNA extraction

The total RNA (5 μg) from the leaves (100 mg per sample) of Gantai-2-2 and Wan82–178 by using TRIzol kit (Invitrogen, Carlsbad, CA, USA) according to the manufacture’s protocol. Briefly 1.3 ml of TRIzol kit was added into 100 mg of leaf sample, the sample was homogenized using power homogenizer and centrifuged at 12,000×g for 10 min at 4 °C. After the fatty layer was removed and discarded, the cleared supernatant was transferred into a new tube and mixed with 0.2 ml of chloroform. The sample tube was shaken for 15 s, followed by an incubation for 3–5 min at room temperature. Next, the sample was centrifuged 12,000×g for 10 min at 4 °C and the aqueous phase was moved into a new tube for RNA precipitation. For precipitating RNA from each sample, 10 *μ*g of RNase-free glycogen was added to the aqueous phase as a carrier, followed by 0.5 ml of 100% isopropanol, then samples were placed at −20 °C for 1 h and centrifuged at 13,600×g for 20 min at 4 °C and discarded the supernatant. To wash the RNA pellet, we added 1.0 ml of 75% ethanol into the tube, vortexed the tube gently, centrifuged the tube 13,600×g for 3 min at 4 °C and discarded the wash. The RNA pellet was air-dried, suspended in 50 μl Nuclease-free water, incubated at 55–60 °C for 10 min. RNA concentration, purity and integrity were determined using a NanoDrop2000 (Thermo Fisher Scientific, Waltham, MA, USA) and an Agilent 2100 Bioanalyzer (Agilent, Santa Clara, CA, USA).

### cDNA library construction and transcriptome deep sequencing

Equal amount of total RNA (5.0 *μ*g) was used for cDNA library construction using TruSeq™ RNA Sample Prep-aration Kit v2 (Illumina) (Illumina, SanDiego, CA, USA) and the cDNA library was sequenced on an Illumina HiSeq™ 2000 platform (Hiseq2000 Truseq SBS Kit v3-HS (200 cycles), Illumina) following the manufacturers’ protocols. Briefly, Dynal Oligo (dT) beads (Invitrogen) were obtained poly(A) mRNAs. Then mRNAs were chemically fragmented into ~200 nt fragments. First-strand cDNA was generated by using reverse transcriptase and random primers, the second strand cDNA synthesis using DNA Polymerase I (Invitrogen) and RNase H (Invitrogen) treatment. The cDNA fragments were end repaired by using End Repair Mix (Illumina) reagent, Finally, purified and enriched to create the final cDNA library. Eight cDNA libraries were sequenced by using pair-end (2 × 100 bp) sequencing technology with an Illumina HiSeq™ 2000 sequencer (The RNA-Seq test and its results were analyzed by the BGI Tech Solutions Co., Ltd. (BGI Tech) (BGI, Shenzhen, PR China).

### Raw read filtering and mapping to the reference genome and gene sequences

SOAPfuse software was used to filter noise for the original sequencing reads [139]. Raw reads of eight libraries were performed by removing adapter sequences and low-quality reads, higher N rate sequences, and excessively short sequences. After passing the QC process of the alignment, the results were used for further analysis. The remaining high-quality reads were submitted for mapping analysis against the soybean reference genome (ftp://ftp.jgi-psf.org/pub/compgen/phytozome/v9.0/early_release/Gmax_275_Wm82.a2.v1/, version Glyma 2.0, 975 Mb), using BWA software and Bowtie software and allowing two base mismatches. We assembled the transcripts with reads using Cufflinks (http://cufflinks.cbcb.umd.edu/) [140].

### DEG analysis

Genes and isoforms expression level are quantified by a software package: RSEMV1.2.12 (RNASeq by Expectation Maximization) [141]. RSEM computes maximum likelihood abundance estimates using the Expectation-Maximization (EM) algorithm for its statistical model, including the modeling of paired-end (PE) and variable-length reads, fragment length distributions, and quality scores, to determine which transcripts are isoforms of the same gene. The expression quantity of each gene (fragments per kilobase of exon model per million mapped fragments, FPKM) was used for the calculated expression level, the formula for which is as follows:

$$ \mathrm{FPKM}\ \left(\mathrm{A}\right)=\frac{10^6C}{N/{10}^3} $$

where FPKM (A) is the expression of gene A, C is the number of fragments that are uniquely aligned to gene A, N is the total number of fragments that are uniquely aligned to all genes, and L is the number of bases on gene A. The FPKM method can eliminate the influence of different gene lengths and sequencing discrepancies in the calculation of gene expression. Therefore, the calculated gene expression can be directly used for comparing the difference in gene expression among samples. According to the Noiseq method, an absolute value of |Log2FC (Fold Change)| ≥ 1 and diverge probability ≥0.8 were used as the threshold to screen DEGs. In the DESeq2 method, the absolute value of |Log2FC (Fold Change)| ≥ 1 and p-adj ≤ 0.001 were used as the threshold to judge the significance of the gene expression difference. For the EdgeR method, the absolute value of |Log2FC (Fold Change)| ≥ 1 and FDR (False Discovery Rate) ≤ 0.001 were used as the threshold to judge the significance of the gene expression difference.

### Bioinformatics analysis

GO (GO, http://www.geneontology.org/) and functional enrichment analysis were conducted on all DEGs using TermFinder software (http://www.yeastgenome.org/help/analyze/go-term-finder). Then, all DEGs were mapped to a pathway in the KEGG database (http://www.genome.jp/kegg/pathway.html) using Blast_v2.2.26 software. A *p*-value was used as ≤0.05 was used as the threshold to judge the significance of the GO and KEGG pathway enrichment analyses.

The transcript version number of Wm82.a2.v1, which was obtained from the sequencing analysis was deleted from the website (http://www.soybaen.org/correspondence/) and only the names of the genes were retained. These names were converted into the differential gene transcription version number of Wm82.a2.v1. MapMan software (http://www.gabipd.de/projects/MapMan/) was used to assign the pathways and functional classifications to the DEGs. The latest Osa_MSU_v7 mapping file and pathways files were downloaded from the website.

### Quantitative real time-PCR (qRT-PCR) analysis

qRT-PCR analysis was used to verify the RNA-Seq results. Primer Premier 5.0 (Premier Biosoft International, Palo Alto, CA) was used to design primers for qRT-PCR experiment (Additional file 13: Table S13). PrimeScript RT Reagent kits with gDNA Eraser (Takara, Dalian, China) were used to reverse transcriptase and synthesize the cDNA. The qRT-PCR reaction mixture (25 μl) contained 12.5 *μ*l SybrGreen qPCR Master Mix (2 × concentration, Ruian Biotechnologies, Shanghai, China), 0.5 *μ*l reverse and forward primers (10 μM), 2 *μ*l cDNA and 9.5 *μ*l ddH_2_O. Then, the qRT-PCR reaction was performed on an ABI7500FAST Real-Time PCR System (Applied Biosystems, Foster City, CA, USA) as follows: 2 min at 95 °C, 40 cycles of heating at 95 °C for 10 s and annealing at 60 °C for 40 s. The *β-actin* gene was used as the internal control to calculate the relative expression. 2^-ΔΔcq^ method was used to calculate the differential expression of a gene in two samples. Three technological and three biological reactions (*n* = 3 × 3) were performed for every gene in each sample.

## Additional files

Additional file 1: Table S1. Total number of DEGs between HRK48 and HRK0. (XLS 163 kb)

Additional file 2: Table S2. Total number of DEGs between HSK48 and HSK0. (XLS 111 kb)

Additional file 3: Table S3. Total number of DEGs between HRK0 and HSK0. (XLS 119 kb)

Additional file 4: Table S4. Total number of DEGs between HRK48 and HSK48. (XLS 101 kb)

Additional file 5: Table S5. GO analysis the DEGs of HRK0-VS-HRK48. (XLS 164 kb)

Additional file 6: Table S6. GO analysis the DEGs of HSK0-VS-HSK48. (XLS 141 kb)

Additional file 7: Table S7. GO analysis the DEGs of HSK0-VS-HRK0. (XLS 137 kb)

Additional file 8: Table S8. GO analysis the DEGs of HSK48-VS-HRK48. (XLS 119 kb)

Additional file 9: Table S9. Pathway analysis the DEGs of HRK0-VS-HRK48. (XLS 108 kb)

Additional file 10: Table S10. Pathway analysis the DEGs of HSK0-VS-HSK48. (XLS 92 kb)

Additional file 11: Table S11. Pathway analysis the DEGs of HSK0-VS-HRK0. (XLS 90 kb)

Additional file 12: Table S12. Pathway analysis the DEGs of HSK48-VS-HRK48. (XLS 90 kb)

Additional file 13: Table S13. DEGs confirmed by the qRT-PCR. (XLS 119 kb)

## Abbreviations

AA: Ascorbic acid
ACC oxidase: Aminocyclopropane carboxylate oxidase
CAD: Cinnamyl-alcohol dehydrogenase
CCOAOMT: Caffeoyl-coA 3-O-methyl transferase
CHI: Chalcone isomerase
CHS: Chalcone synthase
COMT: Caffeic acid 3-O-methyltransferase
CYP: Cytochrome P450
DEGs: Differentially expressed genes
DFR: Dihydroflavonol 4-reductase
DXS: 1-Deoxy-D-xylulose-5-phosphate synthas
ERF1: Ethylene responsive factor 1
ET: Ethylene
GO: Gene Ontology
GST: Glutathione S-transferase
HPT: Homogenitisate phytyltransferas
JA: Jasmonic acid
KEGG: Kyoto Encyclopedia of Genes and Genomes
L-AO: L-ascorbate oxidase
LAR: Leucoanthocyanidin reductase
LOX: Lipoxygenase
MDHA: Monodehy-droaseorbate
OPDA: 12-oxophytodienoic acid reductase
PAL: Phenylalanine ammonia-lyase
POD: Peroxidase
PPO: Polyphenol oxidase
qRT-PCR: Quantitative real time-PCR
ROS: Reactive oxygen species
TRX: Thioredoxin
α-DOX: alpha-dioxygenase
